# Whole methylomes reveal high-altitude-associated methylation at hypoxia and pigmentation genes in South American Indigenous populations

**DOI:** 10.1093/eep/dvaf026

**Published:** 2025-09-24

**Authors:** Yemko Pryor, Nicola Rambaldi Migliore, Daniel Rivas Alava, Rosalinda Di Gerlando, Dean Herman Tineo Tineo, Leonor Gusmão, Fabricio González-Andrade, Alessandro Achilli, John Lindo

**Affiliations:** Program in Genetics and Molecular Biology, Emory University, Atlanta, GA 30322, United States; Department of Biology and Biotechnology ‘‘L. Spallanzani’’, University of Pavia, Pavia 27100, Italy; Department of Anthropology, Emory University, Atlanta, GA 30322, United States; Department of Biology and Biotechnology ‘‘L. Spallanzani’’, University of Pavia, Pavia 27100, Italy; Laboratorio de Biologı´a Forense, Instituto de Medicina Legal y Ciencias Forenses, Ministerio Publico, Lima 15033, Peru; Laboratorio de Diagnostico por DNA (LDD), Universidade do Estado do Rio de Janeiro, Rio de Janeiro 23968-000, Brazil; Translational Medicine Unit, Central University of Ecuador, Faculty of Medical Sciences, Iquique N14-121 y Sodiro-Itchimbia, Sector El Dorado, 170403 Quito, Ecuador; Department of Biology and Biotechnology ‘‘L. Spallanzani’’, University of Pavia, Pavia 27100, Italy; Program in Genetics and Molecular Biology, Emory University, Atlanta, GA 30322, United States; Department of Anthropology, Emory University, Atlanta, GA 30322, United States

**Keywords:** high-altitude Andeans, epigenetic, hypoxia response, UV response

## Abstract

High-altitude adaptation in Andean populations has traditionally been studied through the lens of genetic variation, with limited exploration of epigenetic mechanisms such as DNA methylation. Here, we present the first whole-methylome data comparing Indigenous populations residing in high-altitude regions of the Ecuadorian Andes to those in low-altitude Peruvian Amazon regions bordering the Andes. By leveraging whole-methylome sequencing rather than methylation arrays, we achieved an unprecedented resolution of epigenetic variation, revealing novel insights into altitude-associated adaptations. We identified significant differentially methylated regions in genes involved in hypoxia response and skin pigmentation that differ from patterns previously observed in high-altitude Tibetan individuals [Lin et al. (Genome-wide DNA methylation landscape of four Chinese populations and epigenetic variation linked to Tibetan high-altitude adaptation. Science China Life Sciences 2023;66:2354–69. https://doi.org/10.1007/s11427-022-2284-8.)]. Our findings highlight the influence that altitude-specific environmental pressures, such as hypoxia and ultraviolet radiation, can have on the epigenetic landscapes observed between human populations. Importantly, we uncovered unique regulatory methylation signatures in the hypoxia response pathways of Andean populations, underscoring a distinct epigenetic trajectory compared to other high-altitude groups. This study represents a step forward in understanding Indigenous American genomic plasticity and demonstrates the value of whole-methylome data over methylation arrays in capturing the complex interplay between epigenetics and the environment. These results support a new approach to studying altitude plasticity and underscore the critical role of epigenetics in shaping population-specific cellular responses in Indigenous communities.

## Graphical Abstract

**Graphical Abstract:**
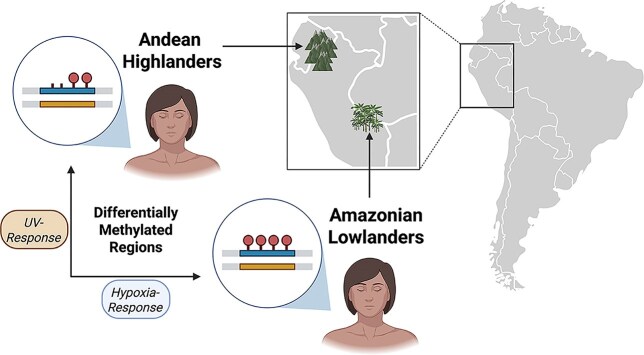
Created in Biorender.com

## Introduction

High-altitude environments (≥2500 meters above sea level—masl) present numerous challenges to human survival and reproduction. These regions are characterized by reduced oxygen levels, increased ultraviolet radiation (UVR), and low ambient temperatures [2–4]. The combination of these extreme environmental conditions and the relatively rapid peopling of the Andes provides a unique opportunity to advance our understanding of human genomic plasticity and cellular responses to high-altitude environments [5, 6]. Archaeological evidence suggests that humans established permanent settlements in the Central Andean Highlands around 9000 years before present [7, 8]. This, coupled with biogeographic evidence of an expansion across the Andean region at approximately the same time, underscores the likelihood of significant adaptation among Andean highlanders and highlights their importance in advancing our understanding of human evolution [7].

Much of the research on the evolutionary history of Andean highlanders has focused on adaptations to the hypoxic high-altitude environment [3, 4, 9, 10]. Native Andean highlanders have demonstrated adaptations to these environmental conditions, including elevated hemoglobin concentrations, larger chest circumferences, and reduced hypoxic ventilatory responses [3, 10, 11]. While recent whole-genome and genome-wide studies have identified adaptive genetic signatures in hypoxia-inducible factor pathway genes, such as *EPAS1* and *EGLN1*, detailed signatures of downstream gene regulatory networks managing cellular plasticity in response to high-altitude—contributing to adaptive phenotypes in Andean populations—remain limited [3, 12, 13, 14].

At high-altitudes, UVR intensity increases by ~9%–11% for every 1000 m of elevation, due to thinner atmospheric conditions that reduce the filtering of UV rays [15, 16]. Skin pigmentation variation, specifically the darkening of skin through the distribution of eumelanin within melanosomes, is a key adaptive phenotype in response to high UVR intensity [17–19]. While reduced eumelanin levels have been associated with human adaptation to higher latitudes outside of Africa, less is known about pigmentation phenotypes in populations migrating back into lower-latitude environments—specifically, Indigenous South Americans living in equatorial regions [18, 20]. Research on the effect of altitude on UVR response among populations at similar latitudes has identified selective signals in several pigmentation genes, including *OCA2* [21, 22]. However, such analyses have yet to include unadmixed Andean populations [23].

While genomic approaches to studying adaptive phenotypes and population-specific selection signals have significantly advanced our understanding of human evolution, investigating epigenetic mechanisms, such as DNA methylation (DNAm), can provide essential regulatory context [24, 25]. This is because epigenetic mechanisms offer insights into gene–environment interactions [24–26]. Research exploring DNAm differences in hypoxia response genes between high- and low-altitude populations has revealed new insights into the regulatory processes that mediate responses to acute versus chronic exposure to hypoxic environments [27]. Similarly, DNAm studies focusing on melanogenesis and population structure have enhanced our understanding of the regulatory mechanisms underlying skin pigmentation genetics [28, 29].

**Figure 1.**
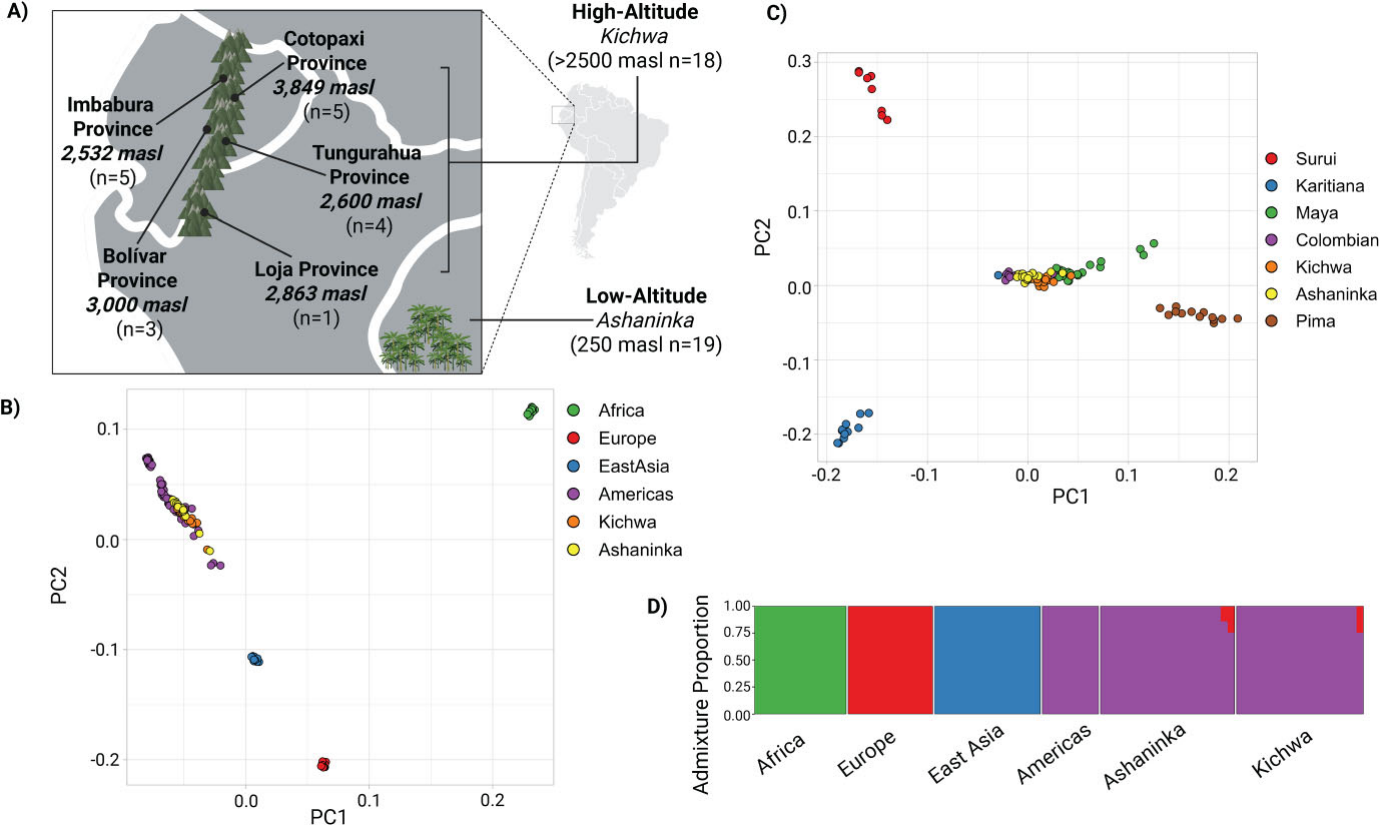
Demographic and ancestry analysis. (A) Map displaying the geographic location and altitude of the populations represented in this study, created with BioRender.com. Altitude is measured in masl. (B) Principal component analysis (PCA) plot of a worldwide dataset including reference populations from the Human Genome Diversity Project (HGDP). Principal components (PCs) were calculated on the HGDP data and the Ashaninka and Kichwa pseudohaploid data (excluding all transitions) were projected onto the HGDP variability. (C) PCA plot including American reference populations from the HGDP. PCs were calculated on the HGDP data and the Ashaninka and Kichwa pseudohaploid data (excluding all transitions) were projected onto the HGDP variability. (D) Supervised ADMIXTURE analysis for the Ashaninka and Kichwa populations at K = 4. Reference populations representing known ancestries were chosen from the HGDP: Mbuti for Africa, Bergamo Italian for Europe, Han for East Asia, and Surui for Indigenous Americans. The ADMIXTURE analysis was run on the pseudohaploid data (no transitions) merged with the HGDP dataset.

In this study, we include both high- and low-altitude Indigenous populations: the Kichwa from the Andean highlands of Ecuador and the Ashaninka from the lowland Amazon Basin along the Peruvian border (Fig. 1A). The lowland Amazon region, characterized by a tropical climate and higher oxygen availability, contrasts sharply with the high-altitude Andes [11]. The Kichwa highlanders live throughout the Andes in Ecuador and speak Quechua, one of the most widely spoken Indigenous languages across the Andes [30]. The Ashaninka lowlanders inhabit the rainforests of the Andean Amazon in Peru, near the border with Brazil [31], and their language belongs to the Arawakan family. Both populations experienced significant declines following the Spanish invasion, partly due to the spread of pathogens [31, 32]. Despite the high levels of indigeneity in both communities, no genetic or epigenetic comparisons between these two populations have been conducted to date [31, 32].

To investigate the population history of high- versus low-altitude Indigenous populations through an epigenetic lens, we present whole-methylome sequencing data from both populations and conduct statistical comparisons of differentially methylated cytosines (DMCs) and differentially methylated regions of DNA (DMRs). To contextualize these findings, we annotate genes within DMRs between the Ashaninka and Kichwa, focusing on those associated with hypoxia and skin pigmentation, followed by gene pathway enrichment analysis. Our goal is to assess whether DMR data can provide insights into the plasticity of cellular phenotypes and inform future studies of human adaptation to both hypoxic and UV-intensive environmental conditions (Fig. S7).

## Results

### Demographic analysis

We sequenced enzymatic methyl-seq (EZ-seq) whole methylomes of 39 individuals, each with a high level of Indigenous American ancestry, from two regions: the high-altitude Ecuadorian Andes (>2500 m) and the low-altitude rainforest near the Peru–Brazil border (Fig. 1A and Table S2). We then assessed relatedness (see the section ‘Methods’) and removed two individuals from two distinct pairs identified as second cousins. The remaining 18 Kichwa individuals come from various provinces within the Ecuadorian Andes (Fig. 1A and Fig. S1), while all 19 Ashaninka individuals are from the Oxapampa province in the Pasco region of Peru. Detailed information on the consent process and agreements with Indigenous communities is provided in the supplement (Figs S2A and S2B).

To determine the level of Indigenous American ancestry within all participants, we performed a pseudohaploid calling, excluding C→T and G→A transitions to avoid errors from cytosine conversion during library preparation (see the section ‘Methods’). Principal Component Analysis (PCA), using global reference populations from the Human Genome Diversity Project (HGDP) (Table S1) [33], revealed that the Ashaninka, Kichwa, and other individuals clustered within the broader set of Indigenous American populations (Fig. 1B). When restricting the analysis to individuals from the Americas, we observed close clustering among the Ashaninka, Kichwa, Colombian, and Maya samples, with these groups remaining distinct from Karitiana, Surui, and Pima populations (Fig. 1C). These patterns indicate high levels of Indigenous ancestry in the Ashaninka and Kichwa, while also reflecting their distinct genetic profiles. To confirm these findings, we ran a supervised ADMIXTURE [34] analysis (Fig. 1D), which identified three individuals with ~25% European admixture. We retained these individuals in downstream analyses, as they still exhibited high Indigenous ancestry and did not drive the observed differential results (see the section ‘Methods’)

### Differential DNA methylation analysis

**Figure 2.**
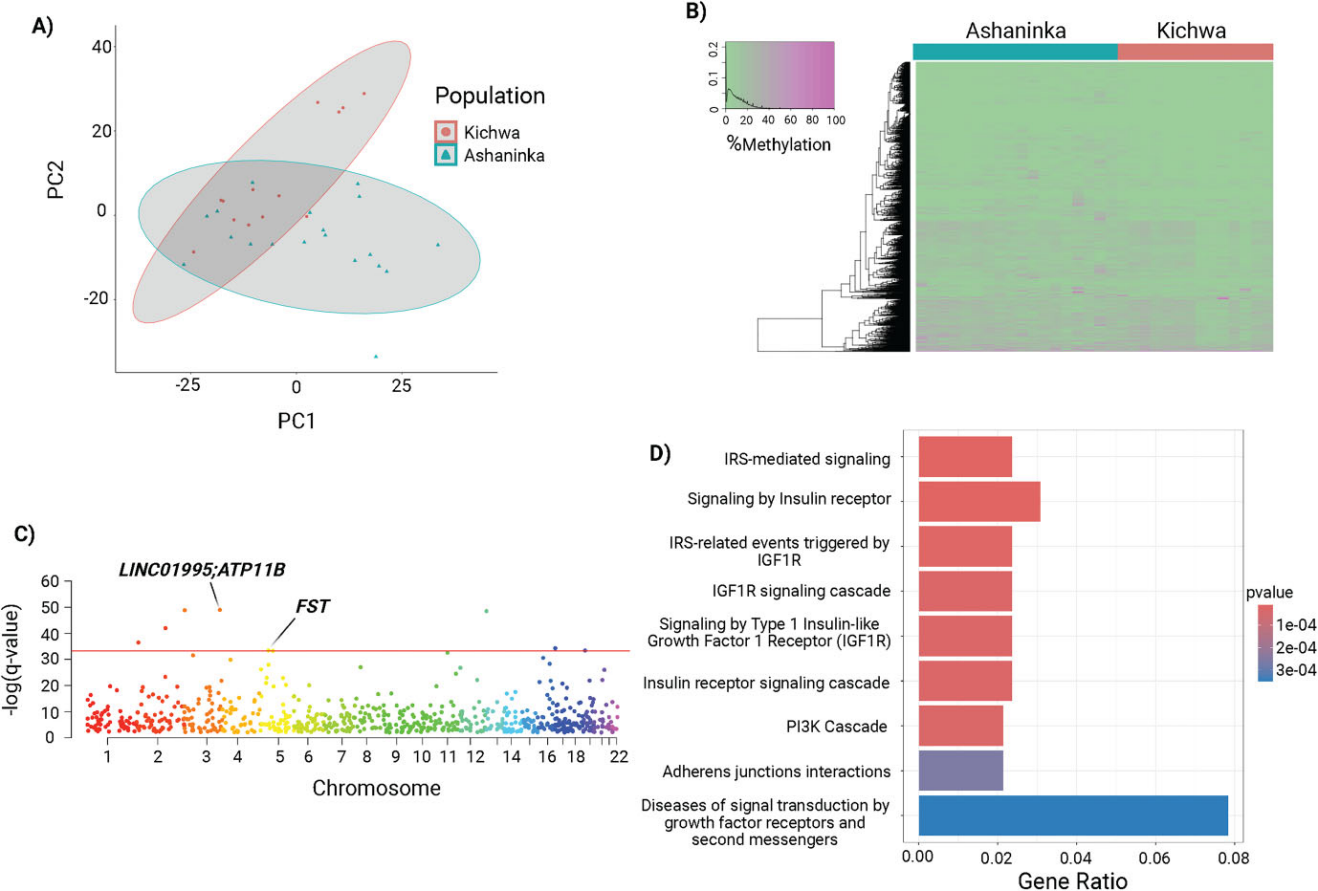
DM analysis: DMRs, genes and pathways of interest. (A) PCA plot showing the first two PCs of the DMC data. (B) Heatmap illustrating differences in the DNA methylation landscape between the Kichwa and Ashaninka populations, based on the DMC data. (C) Manhattan plots of *q*-value and DM significant DMRs (*q* < 0.01, DM >25%). The line indicates the top 1% of DMRs. (D) Gene pathway enrichment analyses of genes within *q*-value and DM significant DMRs (*q* < 0.01, DM >25%).

We used data from the remaining 37 whole-methylomes to identify DMRs. To account for sex, we conducted a sensitivity analysis (Fig. S3) to determine whether sex significantly influenced differential methylation (DM) [35, 36]. Previous studies have shown that sex can impact DNA methylation patterns in various ways. For example, a meta-analysis found widespread differences in CpG methylation between males and females in blood, partly due to processes like X-inactivation, where DNA methylation plays a major role [36]. Since the Ashaninka cohort included only females, we compared the DMR landscape with and without male individuals from the Kichwa group (see the section ‘Methods’) and ultimately chose to exclude all males to control for potential sex-specific methylation differences (Fig. S3). From the remaining 32 whole-methylome samples, we used methylKit [37] to identify a total of 1455 DMCs and 5044 DMRs across the entire DNA methylome, applying the Benjamini–Hochberg (BH) correction method to control for the false discovery rate (FDR) with a threshold of *q* = 0.01. We then performed PCA, which revealed distinct clustering of DMCs between the Ashaninka and Kichwa populations, suggesting that some methylation variation is population-specific (Fig. 2A and Fig. S6). Additional clustering within the Kichwa appears to stem from regional proximity, as the four individuals in the farthest cluster are from the northern regions of the Andes spanning the provinces of Imbabura and Cotopaxi (Fig. S8). The subclusters within the Ashaninka result from previously described genetic subgrouping [31]. The *Ashaninka1* subgroup (largely clustering inside of the overlap in Fig. 2A) likely experienced more isolation as they show longer runs of homozygous DNA fragments [31].

To better understand the distribution of hypermethylation and hypomethylation between the two populations, we generated a heatmap to visualize the % methylation matrix. This showed that most of the methylome is similar between both populations, although the Ashaninka exhibited slightly higher levels of hypermethylation (Fig. 2B). This is consistent with the close tight clustering between the two populations (Fig. 1B and C).

To identify DMRs, we set a minimum read threshold of three reads and normalized coverage across all sites to minimize bias in downstream statistical tests. Given the high genetic similarity between the two populations in this study, we interpreted the data at a range of DM thresholds (Fig. S4) but ultimately defined significant DMRs as those with DM >25% and *q* < 0.01. Previous research highlights how environmental conditions, including altitude, shape the methylome of closely related populations and ethnic subgroups [1, 38]. We identified that our DMR signal was not driven by the three admixed individuals (Fig. S5), so we decided to include them in our downstream analyses.

We identified a total of 779 DMRs meeting our significant criteria (DM >25% and *q* < 0.01). DMRs with negative DM values indicate hypomethylation in the Kichwa compared to the Ashaninka, whereas positive DM values indicate hypomethylation in the Ashaninka relative to the Kichwa.

We examined genes within the top 1% of DMRs that met all significance thresholds (*q* < 0.01, DM >25%) in our dataset (Fig. 2C; Table S3). The most *q*-value significant DMR (*q* = 8.25E-50, DM = –31.12) is located upstream of the Long Intergenic Nonprotein Coding RNA 1995 (*LINC01995*) and ATPase Phospholipid Transporting 11B (*ATP11B*) genes (Table S3). This DMR overlaps an upstream enhancer and an epigenetically modified accessible region. While *LINC01995* is not well characterized, *ATP11B* encodes a member of the P-type ATPase family, with a known role in innate immunity through neutrophil degranulation [39]. Previous research has shown that neutrophil degranulation is increased in hypoxic environments [40].

We identified one hypermethylated DMR in the Kichwa that spanned a promoter region in captured in the top 1% of our analysis. This DMR is within the follistatin (*FST*) gene (*q* = 3.96E-34, DM = 33.340731) (Table S3). In addition to the promoter, this *FST*-associated DMR also spans a CTCF-binding region within the gene’s first intron, suggesting potential regulatory and chromatin-organizing functions.

### Gene ontology analysis

All genes within the 5044 *q*-value significant (*q* < 0.01) DMRs were further analysed to uncover potential patterns and pathway enrichment using the ReactomePA R package [41]. The results indicate a high prevalence of genes involved in neurological pathways and disease-related processes, particularly through growth factor receptors and secondary messengers (Fig. S11). This is likely linked to the multiple roles of HIF-1⍺, a subunit of the heterodimeric protein HIF-1, that plays a critical role in directing cellular response to hypoxia through the transcriptional regulation of hypoxia response elements (HREs). Under normoxic conditions in the neuronal system, HIF-1⍺ is involved in many processes, including its regulation of both cell survival and death pathways, its expression in glial cells under normoxic conditions, and its ability to upregulate growth factors, receptors, and other signaling proteins [42, 43].

Interestingly, a proportion of the DMR genes are involved in the PI3K/AKT signalling pathway (Fig. 2 and Fig. S11). Modulation of HIF-1⍺ activity through this pathway has been previously observed, particularly under hypoxic conditions linked to certain types of tumour progression [44–46, 47]. Additionally, follistatin-induced skeletal muscle hypertrophy has shown an association with PI3K/AKT signalling [48]. Regulation of skeletal muscle growth and development is especially important under hypoxic conditions, where this growth is often impaired [49, 50]. The enrichment plot reflects overlap between gene sets involved in both the PI3K/AKT pathway and growth factor receptors, suggesting that these molecular processes may be influenced by the differences in altitude at which these two closely related populations reside.

To further investigate the pathways enriched within our DMR results, we also performed pathway enrichment analysis on genes within significant DMRs (*q* < 0.01) satisfying the >25% DM threshold (Fig. 2E). The top pathways represented included genes interfacing with insulin receptors and growth factors, many of which activate the PI3K/AKT pathway [51, 52]. IGF1R activity has been found to help increase skeletal muscle growth, working in tandem with follistatin [53]. Additionally, knockdown of IGF1R or IGF-1 activity has previously been shown to ameliorate the effects of chronic hypoxia-induced pulmonary hypertension [54]. Changes in IGF-1 have also been shown to modulate the activity of HIF-1 and VEGF, impacting vascular growth [55]. IGF-1 activity has also been found to influence the differentiation of precursor cell lineages into melanocytes during development [56].

Given our interest in traits related to hypoxia and UV response, we utilized Reactome along with specialized databases of pigmentation and UV-response genes to further investigate our DMR results (Tables S5 and S6) [39, 57–60]. We identified two DMRs spanning genes related to cellular response to hypoxia, satisfying all significance parameters (*q* < 0.01, DM >25%). The hypoxia response-related genes associated with these DMRs were *RBX1* and *PSMA8* (DM = 38.6% and 40.6%; *q* = 1.61E-12 and 5.27E-06, respectively) (Table 1). The *RBX1* gene encodes the Ring-Box 1 protein, a component of the pVHL-E3 ligase complex that regulates HIF-1⍺ under normoxic conditions [61, 62]. The *PSMA8* gene encodes the 26S proteosome, which exhibits a hypoxia-induced decrease in activity [63]. Similarly, *PSMA8* encodes a subunit of the 26S proteasome, whose activity decreases under hypoxic conditions. Inhibition of the 26S proteasome in normoxic environments has been shown to increase endogenous HIF-1⍺ protein levels [64]. The DMR near *RBX1* overlaps a distal promoter ~90 kilobases (kb) downstream of *RBX1*. For *PSMA8*, the nearest DMR is located ~4 kb distally from *PSMA8* and sits between two enhancers that are both ~5 kb outside the DMR. All five genes exhibited hypomethylation in the Kichwa population, suggesting potential differences in gene expression between the Kichwa and Ashaninka, as DNA methylation is known to influence transcriptional regulation [65].

**Table 1.** Top DM and FDR-significant DMRs associated with hypoxia response pathway genes.

CHR (hg19)	Start	End	Gene	MethylKit *P*-value	MethylKit *q*-value	Methylation difference	Reactome pathway
Chr 22	41 460 014	41 460 272	*RBX1* (distance = 90 701)	9.87E-14	1.61E-12	−38.623207	Cellular response to hypoxia
Chr 18	23 777 436	23 777 673	*PSMA8* (distance = 4117)	1.47E-06	5.27E-06	−40.602234	Cellular response to hypoxia

Negative methylation differences indicate hypermethylation in the Ashaninka, while positive values indicate hypermethylation in the Kichwa. All DMRs listed in this table satisfy all significance parameters (DM <25%, *q* > 0.01).

When scanning for genes related to UVR response and skin pigmentation, we identified 39 pigmentation-associated genes within FDR-significant DMRs (*q* < 0.01). These included the Epidermal Growth Factor Receptor (*EGFR*) and the OCA2 Melanosomal Transmembrane Protein (*OCA2)* genes, which overlapped within DMRs satisfying all significance metrics (DM >25% and *q* < 0.01) [57–60]. *EGFR* (DM = 26.1%, *q* = 1.77E-06) displayed hypomethylation in the Kichwa (Table 2). This DMR is in a promoter region ~85 kb upstream of *EGFR*. Previous studies have associated alleles in this gene with skin pigmentation variation in admixed Indigenous populations from both North and South America [66]. Additionally, one DMR located within *OCA2* (DM = 66.7%, *q* = 2.04E-06) spanned an intron of the gene and overlapped with ~250 bp of a 375-bp length of open chromatin. DNA methylation differences in these genes have been linked to pigmentation variation in European populations and human vitiligo cell lines [67, 68]. In our analysis, the *OCA2-*related DMR was hypermethylated in the Kichwa compared to the Ashaninka (Table 2). These findings suggest the presence of unique and previously undetected patterns of pigmentation regulation and variation between high- and low-altitude Indigenous populations.

**Table 2.** Top DM and FDR-significant DMRs associated with pigmentation genes.

CHR (hg19)	Start	End	Gene	Methylkit *P*-value	Methylkit *q*-value	Methylation difference	Reactome pathway
Chr 9	89 226 598	89 226 957	*GAS1 (distance = 332 320)*	9.40E-12	1.03E-10	−32.63301011	Signal transduction
Chr 10	123 489 217	123 489 495	*FGFR2 (distance = 131 245)*	9.93E-11	8.96E-10	−32.97571993	PIP3 activates AKT signaling
Chr 5	77 590 137	77 590 721	*AP3B1*	2.83E-07	1.23E-06	−28.6194699	Golgi-associated vesicle biogenesis
Chr 7	55 000 876	55 001 135	*EGFR (distance = 85 575)*	4.25E-07	1.77E-06	−26.11711449	PTK6 promotes HIF1A stabilization
Chr 15	28 147 925	28 148 465	*OCA2*	4.99E-07	2.04E-06	66.66 666 667	Melanin biosynthesis
Chr 3	158 551 099	158 551 377	*MFSD1 (distance = 3591)*	0.000 133 719	0.000 278 924	−26.53061224	GPCR downstream signaling
Chr 6	56 498 046	56 498 362	*DST*	0.000 158 031	0.000 322 653	−47.16981132	Assembly of collagen fibrils and other multimeric structures
Chr 9	20 097 800	20 098 040	SLC24A2 *(distance = 308 765)*, MLLT3 *(distance = 243 627)*	0.000 559 934	0.000 962 731	−30	Sodium/calcium exchangers; formation of RNA Pol II elongation complex

Negative methylation differences indicate hypermethylation in the Ashaninka, while positive values indicate hypermethylation in the Kichwa. All DMRs listed in this table satisfy all significance parameters (DM <25%, *q* > 0.01).

## Discussion

Both genetic and epigenetic factors have been extensively studied in the context of high-altitude adaptation in Andean populations [10, 11, 27, 69, 70]. While genetic data capture the long-term evolutionary history and natural selection processes within populations, the study of epigenetic mechanisms offers additional insights into how adaptive phenotypes may respond dynamically to physiological and environmental changes [65, 71]. Our findings showcase the interplay between epigenetic regulation and hypoxia response-related genes, such as *PSMA8* and *FST*, and highlight additional pathways, including the PI3K/AKT pathway, as areas for further exploration. In addition, we identified 39 pigmentation-associated genes within our *q*-value significant DMRs, suggesting that altitude might also influence skin pigmentation variation even among closely related populations.

One of our major findings suggests that DNAm variation within genes associated with PI3K/AKT pathway regulation may be influenced by chronic hypoxic environments. PI3K/AKT activation has been correlated with follistatin activity in promoting skeletal muscle hypertrophy [48]. This pathway has been studied extensively in the context of cancer, as hypoxic tumour environments can activate HIF-1, the heterodimeric protein consisting of the HIF-1⍺ and HIF-1β subunits, to promote cell proliferation by increasing glycolysis [72] and vasodilation [73]. Interestingly, PI3K/AKT pathway activity has been shown to influence both glycolysis and the transcriptional activity of HIF-1 [74]. HIF-1 activation of downstream targets like Vascular Endothelial Growth Factor (*VEGF*) could further modulate follistatin activity, as previous research has shown an upregulation of *FST* upon *VEGF* treatment [75]. In addition, the PI3K/AKT pathway has been implicated in arteriole wall thickening under hypoxic conditions in rats, as well as human cells [76–78]. Arteriole wall thickening in humans has also been linked to the development of pulmonary hypertension, which is more common amongst Andean highlanders compared to other highland populations [10, 79]. Similar adaptations have been observed in high-altitude Andean populations, including increased muscularization of small arteries and higher blood viscosity [80, 81], which may represent a unique vascular adaptation distinct from those found in Tibetan populations [2].

The DMR spanning the *FST* promoter also stood out as a major finding. Follistatin inhibits the secretion and synthesis of the follicle-stimulating hormone and regulates skeletal muscle development through its activity as an activin A and myostatin antagonist, respectively [48, 82]. Activins and myostatin are members of the transforming growth factor-beta superfamily, which are involved in several biological processes including inflammation, skeletal muscle growth, reproduction, and developmental biology [48, 82]. Follistatin has previously been shown to be downregulated under hypoxic conditions in endothelial cells [83]. This is interesting, given that our results indicate hypermethylation of the DMR spanning *FST* (Table S3), which could suggest downregulation of *FST* activity in the Kichwa compared to the Ashaninka. Additionally, previous research has described the presence of right ventricular hypertrophy (RVH) in Andeans living and low and high-altitude, demonstrating persistence of slight RVH into adolescence in highlanders compared to lowlanders [84]. This is interesting, given that previous research has also described how variation in the delicate balance between follistatin/myostatin activity likely facilitates cardiac hypertrophy [85]. Furthermore, the higher prevalence of pulmonary hypertension in Andean highlanders compared to other highlanders also frames potential impacts of this *FST DMR*, given that *FST* regulation has been shown to protect against hypoxia-induced pulmonary hypertension in mice [86–88]. These findings underscore a link between DNA methylation and the modulation of cellular plasticity in response to hypoxic stress in high-altitude Andeans.

Previous studies in high-altitude populations, including Andeans and Tibetans, have identified other hypoxia-related genes, such as *EGLN1* and *EPAS1* [10, 89]. Both *EGLN1*, which encodes PHD2, and *EPAS1*, which encodes the HIF-2⍺ subunit have been linked to high-altitude adaptation in both Andean and Tibetan populations, while [12, 90, 91]. Although HIF-1⍺ and HIF-2⍺ are isoforms with slightly different regulatory functions, both act as transcription factors in conjunction with their β-subunits, promoting gene expression by binding to HREs [92]. Studies have shown that high-altitude populations tend to exhibit lower DNA methylation in *EPAS1*-related DMRs, while increased *EGLN1* methylation has been observed in high-altitude Tibetans compared to low-altitude Chinese populations [1, 93]. Interestingly, we were able to identify an FDR-significant DMR overlapping *HIF1AN* (DM = 7.6%, *q* = 2.00E-84) (Table S5), a gene that plays a critical role in the hypoxia response pathway by encoding a suppressor of HIF-1α that inhibits the transcription of HREs under normoxic conditions [94]. Though the *HIF1AN* DMR did not satisfy all significance parameters, our findings of *HIF1AN* hypomethylation, in addition to *PSMA8*, and *RBX1* hypomethylation, may reflect alternative regulatory mechanisms varying cellular responses to chronic hypoxia unique to the high-altitude Andes.

In terms of UVR response, we identified 39 pigmentation-related genes within our FDR significant DMRs (*q* < 0.01) (Table 2). Among them were *OCA2* (DM = 66.7%, *q* = 2.04E-06)*, TYRP1* (DM = 14%, *q* = 7.70E-07), and *ASIP* (DM = 11.8%, *q* = 0.003), which have been linked to DNA methylation differences associated with pigmentation variation. Additionally, the Opioid Receptor Mu 1 (*OPRM1*) (DM = −10.1%, *q* = 5.75E-10) and *EGFR* (DM = 26.1%, *q* = 1.77E-06), previously associated with pigmentation in Indigenous populations from the Americas [66], were also found within our significant DMRs. While the influence of altitude on pigmentation and the role of epigenetic mechanisms like DNA methylation remain understudied [95, 96], one genome-wide study comparing Tibetan and Han Chinese populations identified 13 pigmentation genes with strong signals of divergent selection, including *OCA2* [71].

Our findings reveal that *OCA2* exhibits a 66.7% DNAm difference between the Kichwa and Ashaninka populations, suggesting that epigenetic regulation of this gene may play a role in skin pigmentation variation between highland and lowland Indigenous populations. These results provide a promising avenue for further research on the interplay between altitude, pigmentation, and epigenetic regulation, and offer insights into the divergent elements contributing to skin pigmentation variation among closely related populations living at similar latitudes.

Although compelling, our study is limited by the lack of a high–low altitude ancestral migration comparison. Currently, we are not able to discern whether the Ashaninka would exhibit similar DMR phenotypes if they lived at high-altitude. This information is needed to determine whether or not Andean highland ancestry could be influencing DMR phenotypes. Previous studies of Andean populations have compared genome-wide DNA methylation profiles among three groups with high-altitude Quechua ancestry: lifetime, limited, and no high-altitude exposure [3]. These studies identified DMRs and DMCs associated with developmental exposure to high-altitude environments. Notably, shared epigenetic signatures were observed among Andean highlanders even after limited high-altitude exposure. These results highlight the long-term effects of chronic hypoxia during development and the potential role of epigenetic mechanisms in guiding adaptive phenotypic plasticity [3]. Future research improving upon the current study design by including individuals with high-altitude Kichwa ancestry living at low-altitude, and individuals with low-altitude Ashaninka ancestry living at high-altitude could allow evaluation of whether ancestry impacts epigenetic variation in response to high-altitude environments. These types of data would greatly bolster our ability to determine whether highland ancestry, along with hypoxic environments, together influence potential instances of adaptive phenotypic plasticity to high-altitude.

Applying whole-methylome sequencing technologies to a similar study design would substantially advance our understanding of the interplay between DNA methylation and adaptive phenotypes, helping to further unravel the evolutionary history of life in the high-altitude Andes. In addition, the lack of representation of non-European populations in reference datasets is a major limitation when attempting to identify confounders, such as cell type composition and biological age using currently available computational tools and array-based platforms (Figs S9 and S10). The data and limitations presented in this study underscore the urgent need for more equitable representation of Indigenous populations in population genetics and epigenetics research.

### Limitations of the study

Whole-methylome and whole-genome approaches are essential for comprehensively investigating human genetic and epigenetic diversity. However, the high cost of sequencing at this scale has limited the number of individuals included in this study, as well as the sequencing coverage (Table S2). While this study provides a foundation for future research, expanding its scope will require the incorporation of additional molecular biology techniques, including both *in vivo* and *in vitro* methods. These approaches will enable researchers to explore the genetic and epigenetic interactions identified here under controlled conditions and in model systems. Such efforts will be crucial for gaining deeper insights into the mechanisms by which Andean highlanders have uniquely adapted to life at high-altitude.

## Methods

### DNA sample collection

The collection of DNA samples for this study was carried out with the support of Indigenous peoples in Peru and in partnership with Indigenous communities in northern Ecuador [31]. Written informed consent was obtained from all participants, ensuring confidentiality and voluntary participation. To protect the privacy of the communities represented in this research, no health or immune status data will be disclosed.

*Kichwa: high-altitude population*. DNA from donor blood was extracted using FTA cards from the high-altitude Kichwa individuals. The blood samples were collected in collaboration with Indigenous communities from the Ecuadorian Andean provinces of Cotopaxi, Tungurahua, Bolívar, Loja, and Imbabura, as detailed in a previous publication [32]. All donors participated voluntarily, signing informed consent forms (Fig. S2A), and their samples were deidentified to ensure privacy.

*Ashaninka: low-altitude population*. The collection of biological samples and DNA extraction from individuals self-identifying as Ashaninka in the Pasco region of Peru were conducted as part of previous studies. DNA was extracted from bloodspots using a common phenol–chloroform protocol [97]. Informed written consent was obtained from all Ashaninka participants, and a scientific agreement was established with the Indigenous community (Fig. S2B), as described in prior publications [31, 97, 98].

### Initial data preparation

For the DNA extraction from Kichwa blood samples, we used the Qiagen QIAamp DNA Investigator Kit, following the modifications specific to FTA and Guthrie cards. Biological samples from individuals self-identifying as Ashaninka in the Pasco region of Peru were collected as part of previous studies and DNA was extracted from blood spots using a standard phenol–chloroform protocol [31, 97, 98]. Following extraction, DNA quality and concentration were assessed using a Thermo Fisher Qubit Fluorometer.

### Enzymatic methyl-sequencing and bioinformatics

Enzymatic methyl-sequencing (EM-seq) libraries were built for the 41 individuals using the NEBNext EM-Seq Kit (New England Biolabs) for identification of both 5-mc and 5-hmc, which outputs higher quality libraries than traditional bisulfite conversions techniques [99]. The library quality and concentration were evaluated using the Thermo Fisher Qubit Fluorometer and Agilent DNA High sensitivity TapeStation. All libraries passed initial QC with concentrations over 2 ng/µl and average base pair lengths between 300 and 450 bp and were sequenced at a mean coverage of 7.5x on an Illumina Novaseq 6000 (Psomagen). Data from 39 of the 41 individuals was returned, as two were removed. Sequenced reads were aligned to hg19 methylome using Bismark [100], utilizing best practices and removing duplicates.

To assess familial relationships within the dataset, we utilized two methods from VCFtools: AJK-statistic (relatedness) and the KING (relatedness2) [101, 102]. KING estimates kinship coefficients while correcting for population structure, making it robust in diverse populations, and the AKJ-statistic evaluates relatedness through allele-sharing patterns, providing estimates of IBD0 (no shared alleles) and IBD1 (one shared allele). Using these tools, we identified related individuals by applying established thresholds: R ≥ 0.45 for first-degree relatives (e.g. parent–offspring, full siblings) and 0.20 ≤ R < 0.45 for second-degree relatives (e.g. half-siblings, cousins). We excluded one individual from each related pair, leaving 37 individuals, prioritizing higher sequencing coverage to maintain dataset independence. This dual-method approach ensured robust detection and exclusion of related individuals, minimizing confounding effects in downstream analyses.

### Ancestry analysis

Given the low coverage of the data (Table S2) and base changes due to methyl-seq enzymatic conversion, we performed a pseudohaploid calling using the *pileupcaller* command in SequenceTools [103] after removing all transitions. Sites were called on the HGDP [33] variant dataset, using the PASS filter to select single nucleotide polymorphisms (SNPs). These SNPs were then lifted over the GRCh37 reference with the picard-tools [104], *LiftOverVcf*, command. Variants were first filtered using PLINK [105] for *–geno* 0.05 *and –maf* 0.05 before using them for the peudohaploid calling, leaving a total of 6 024 373 SNPs. The resulting number of called transversions in our dataset ranged from 118 013 to 1 391 148. After performing the pseudohaploid calling described above, we merged our data with a subset of the HGDP [33], including the following populations: BergamoItalian, Han, Mbuti, Colombian, Maya, Pima, Karitiana, and Surui.

To confirm high levels of Indigenous American ancestry, we performed PCA using Eigensoft [106] *smartpca* with the parameters *lsqproject*: YES, *numoutlieriter*: 0, *shrinkmode*: NO, and *numoutevec*: 10, computing the principal components (PCs) on the HGDP populations and projecting our individuals on the PC space. To identify ancestral relationships between the Ashaninka and Kichwa compared to global populations, we first ran the PCA with the following populations from the HGDP [33]: BergamoItalian, Han, Mbuti, Colombian, Maya, Pima, Karitiana, and Surui. Then, to assess ancestral relationships between populations in the Americas specifically, we ran an additional PCA with the Ashaninka, Kichwa, and the following populations from the HGDP: Colombian, Maya, Pima, Karitiana, and Surui.

We then performed a supervised ADMIXTURE [34] analysis. The dataset was filtered using PLINK [105], for missingness (*—geno 0.25*) and to remove SNPs in linkage disequilibrium (*—indep-pairwise 200 25 0.4*) for a final set of 248 978 SNPs. ADMIXTURE was run with the *–supervised* using BergamoItalian, Han, Mbuti, and Surui as reference populations (Table S1).

### DNA methylation and ontology analysis

Bismark-generated bedgraph files were loaded into R for analysis via Methylkit [37, 100]. Methylkit is an R package that rapidly analyses high-throughput DNA methyl-sequencing data, using logistic regression and Fisher’s exact test to identify DMRs [37]. Using the *methRead* function, BAM files were read and annotated with two conditions, low altitude (control) and high-altitude (treatment) [37]. Data filtration parameters were set to a minimum read count of three, and all reads exceeding 99.9% coverage in each sample were removed with the *filterByCoverage* function [37]. To account for the skewed coverage difference between our populations of interest, all remaining sites were normalized for coverage with a size factor derived from the median ratio method using the *normalizeCoverage* function [37]. After filtration and normalization, all residual sites were merged with *unite* and included in downstream analysis [37]. Using the % methylation matrix calculated within Methlykit via pairwise correlation comparisons, we conducted further cluster analyses. This included PCA (Fig. 2A and Fig. S6), as well as visualization of hierarchical clustering with heatmaps of the DMCs (Fig. 2B). With *calculateDiffMeth*, we identified DMRs at a minimum methylation difference percentage of 5% (Fig. 2D), 10%, 25% (Fig. 2C), and 50% using logistic regression and a BH-adjusted significance threshold of *q* = 0.01 to correct for multiple testing [37] (Fig. S4 and Supplementary data). Additionally, we conducted a sensitivity analysis comparing the methylome landscape before and after removing the males to control for sex. After proceeding will all females, all data from the remaining 32 individuals were filtered and normalized using the same metrics described above and the differential CpG methylation was again calculated using the *calculateDiffMeth* function [37]. This resulted in a 5% increase in total DMCs from 1388 to 1455, and a 14% decrease in the total number of DMRs, leaving us with 5044 from 5835 significant DMRs (*q* < 0.01) among females only (Fig. S3). Since we observed three admixed individuals in our dataset, we reran Methylkit, excluding these three individuals using the same parameters described above. This yielded a 7% decrease in the DMRs from 5044 to 4712, and a 1% increase in DMCs. We found that our signal was not driven by the admixed individuals after comparing the DMR results before and after removal of the admixed individuals (Fig. S5), and kept them in all downstream analyses. Annovar was then used to annotate the genes and functions represented in our DMRs results [107]. This was followed by the application of the Reactome R package to conduct gene pathway enrichment analysis comparing the genes within the 5044 DMRs at DM >5% vs. the 799 DMRs at DM >25% thresholds (Fig. 2E and F) [41]. All pigmentation-associated genes were identified by overlapping the gene list from our significant DMRs with a list of 688 pigmentation genes from International Federation of Pigment Cell Societies website and the HIrisPlex-S Eye, Hair, and Skin Color DNA Phenotyping Webtool [57–60].

Additional covariates, including age, estimated cell type proportions, and batch effects, were considered during our analysis. We used the *assocComp* function in methylKit to identify PCs potentially correlated with batch effects, as EM-seq libraries were constructed over multiple days [37]. While some correlation between PCs and batch effects was observed, this is likely attributable to population stratification, as library preparation batches were largely organized by subpopulation (Fig. S8). As a result, what appears to be a batch effect may reflect underlying population structure rather than technical variability.

Age information was not collected at the time of initial biological sampling, and per our original consent agreement, we are not authorized to retrieve additional biological or demographic data from individuals in either community. Due to this limitation, we estimated biological age using the methylclock R package [108], which employs a Bayesian network to predict age from CpG methylation patterns, based on nine reference datasets. Although *methylclock* can impute missing data, it recommends that at least 80% of CpG sites be present to ensure optimal accuracy. Our dataset had low overlap with the CpG sites used in these reference datasets, likely due to two factors: (1) the individuals in our study are Indigenous Americans, who are underrepresented in publicly available training datasets, and (2) our data derive from whole-genome DNA methyl-sequencing, whereas the reference datasets used in *methylclock* are based on Illumina 27K, 450K, and EPIC arrays.

As a result, we were only able to train our dataset against the smallest reference model available (Hannum clock), and we lowered the minimum CpG site threshold to 20%, with the remaining CpGs imputed [108–110]. These constraints likely reduce the reliability of the age estimates. Despite this, we found average predicted ages of 39.27 years for the Kichwa and 43.65 years for the Ashaninka (Fig. S9). Although we completed downstream analyses in methylKit using these estimates, we ultimately excluded age-restricted models from our final analysis due to the high likelihood of bias introduced through imputation [37, 108].

To address potential biases arising from variation in cell subtype proportions in whole blood, we attempted deconvolution analysis using the deconvR program [111]. This tool estimates cell-type proportions from whole-methylome data by referencing the Comprehensive Human Methylome Atlas, which includes ~6000 CpGs across 25 cell types [111, 112]. Using the BSMeth2Probe function, deconvR allows users to input methylation data as a methylKit object and map CpGs to probe IDs from the Infinium MethylationEPIC v1.0 B5 Manifest. Like other deconvolution methods, this approach depends on CpG sites targeted by array-based platforms (e.g. Illumina 450K or EPIC arrays), which represent only a small fraction of the genome. The CpGs captured in our whole-genome methylation data do not consistently overlap with those in these reference datasets, resulting in insufficient coverage for reliable deconvolution. Nevertheless, we attempted to map the 1455 CpGs in our dataset to EPIC array probes and obtained cell-type proportion estimates. However, these results are likely inaccurate, as indicated by the unexpectedly high representation of prostate cell types in our female-only cohort (Fig. S10).

### Community engagement and dissemination of results

The results of this study are summarized in Fig. S7. This poster will be translated into Spanish and presented in person to communities in the Tungurahua province (Ecuador) and the Pasco region (Peru) as part of special workshops. The findings will also be shared at the Annual Ecuadorian Congress of Cultural Anthropology.

Workshop Procedure:

The workshops will last ~2 h.

- First 30 min: This section will follow a think-pair-share activity, where participants will form small groups to discuss how they believe altitude impacts human physiology and share personal experiences or observations. Each group will report their insights to the audience, facilitating a broader discussion on the physiological effects of high-altitude environments. This will be followed by a brief PowerPoint presentation framing these responses within an evolutionary context. Participants will then reassemble into small groups to brainstorm how Andean populations might have adapted to high-altitude conditions over time.
- Next hour: A PowerPoint presentation will summarize current knowledge on Andean high-altitude adaptation and Indigenous American genetic history. This section will also provide an overview of the research methods used in this study. Key research questions, methodologies, and findings will be explained, with a focus on the panels presented in Fig. S7. A large-scale printed version of the poster will be on display for participants to reference throughout the discussion.
- Final 30 min: The workshop will conclude with a question-and-answer session, followed by a group activity, where participants will create their own graphical abstract to summarize their understanding of the presentation and the concept of high-altitude adaptation in Andean populations.

Language: All information presented will be translated into Spanish to ensure accessibility.

## Supplementary Material

dvaf026_Supplemental_Files

## Data Availability

The methylation data, in the form of BedGraph files aligned to hg19 for each individual, is available in the NCBI SRA archive under the accession number PRJNA1174728.
