# Supplementary material for: Whole methylomes reveal high-altitude-associated methylation at hypoxia and pigmentation genes in South American Indigenous populations
Source: Environ Epigenet. 2025 Sep 24;11(1):dvaf026. doi: 10.1093/eep/dvaf026 (PMC12542823; doi:10.1093/eep/dvaf026)
Supplement: dvaf026_Supplemental_Files [file dvaf026_supplemental_files.zip › Supplemental_Round2_Revision.docx]

**Supplemental Information**

**Whole Methylomes Reveal High-Altitude–Associated Methylation at Hypoxia and Pigmentation Genes in South American Indigenous Populations**

Yemko Pryor^1^, Nicola Rambaldi Migliore^2^, Daniel Rivas Alava^3^, Rosalinda Di Gerlando^2^, Dean Herman Tineo Tineo^4^, Leonor Gusmão^5^, Fabricio González-Andrade^6^, Alessandro Achilli^2*^ and John Lindo^1,3*^

^1^Graduate Program in Genetics and Molecular Biology, Emory University, Atlanta GA, 30322 USA

^2^ Department of Biology and Biotechnology ‘‘L. Spallanzani’’, University of Pavia, Pavia 27100, Italy

^3^ Department of Anthropology, Emory University, Atlanta GA, 30322 USA

^4^Laboratorio de Biologı´a Forense, Instituto de Medicina Legal y Ciencias Forenses, Ministerio Pu´ blico, Lima 15033, Peru

^5^Laborato´ rio de Diagno´ stico por DNA (LDD), Universidade do Estado do Rio de Janeiro, Rio de Janeiro 23968-000, Brazil

^6^Translational Medicine Unit, Central University of Ecuador, Faculty of Medical Sciences, Iquique N14-121 y Sodiro-Itchimbia, Sector El Dorado, 170403 Quito, Ecuador

*Corresponding authors: [alessandro.achilli@unipv.it](mailto:alessandro.achilli@unipv.it), [jlindo@emory.edu](mailto:jlindo@emory.edu)

**Table of Contents**

Supplementary Figure 1 2

*Kichwa Province and Altitude Information*

Supplementary Figure 2 3

*Kichwa Volunteer Consent Forms*

Supplementary Figure 3 6

*Ashaninka Volunteer Consent Forms*

Supplementary Figure 4 7

*Differential Methylation Analysis: Sex Differences Sensitivity Analysis*

Supplementary Figure 5 7

*Differential Methylation Analysis: Admixture Sensitivity Analysis*

Supplementary Figure 6 8

*Differential Methylation Analysis: DMC PCA Plots*

Supplementary Table 1 9

*Demographic Analysis: HGDP Reference Populations*

Supplementary Figure 7 11

*Community Engagement: Result Dissemination Poster*

Supplementary Table 2 13

*Kichwa & Ashaninka IDs, Altitudes & Methyl-seq Coverages*

Supplementary Table 3 14

*Differential Methylation Analysis:* *Top 15 DM & FDR Significant DMRs*

Supplementary Table 4 14

*Differential Methylation Analysis:* *Top 1% of FDR Significant DMRs at 5% Differential Methylation Threshold*

Supplementary Figure 8 16

*Differential Methylation Analysis: Batch Effects Estimation*

Supplementary Figure 9 17

*Differential Methylation Analysis: Age Estimation Sensitivity Analysis*

Supplementary Figure 10 17

*Differential Methylation Analysis: Cell-Type Deconvolution Analysis*

Supplementary Table 5 18

*Ontology Analysis: All Hypoxia Response-Related Genes within FDR Significant DMRs*

Supplementary Table 6 19

*Ontology Analysis: All Pigmentation-Associated Genes within FDR Significant DMRs*

Supplementary Figure 11. 21

*Pathway Enrichment Analysis of all FDR Significant DMRs at 5% Differential Methylation Threshold*

Supplementary References 21

*Works Cited*

**Supplementary Figure 1:** The Kichwa communities (**bolded**) included in this study, and the provinces and altitudes at which they live. Created with BioRender.com

**
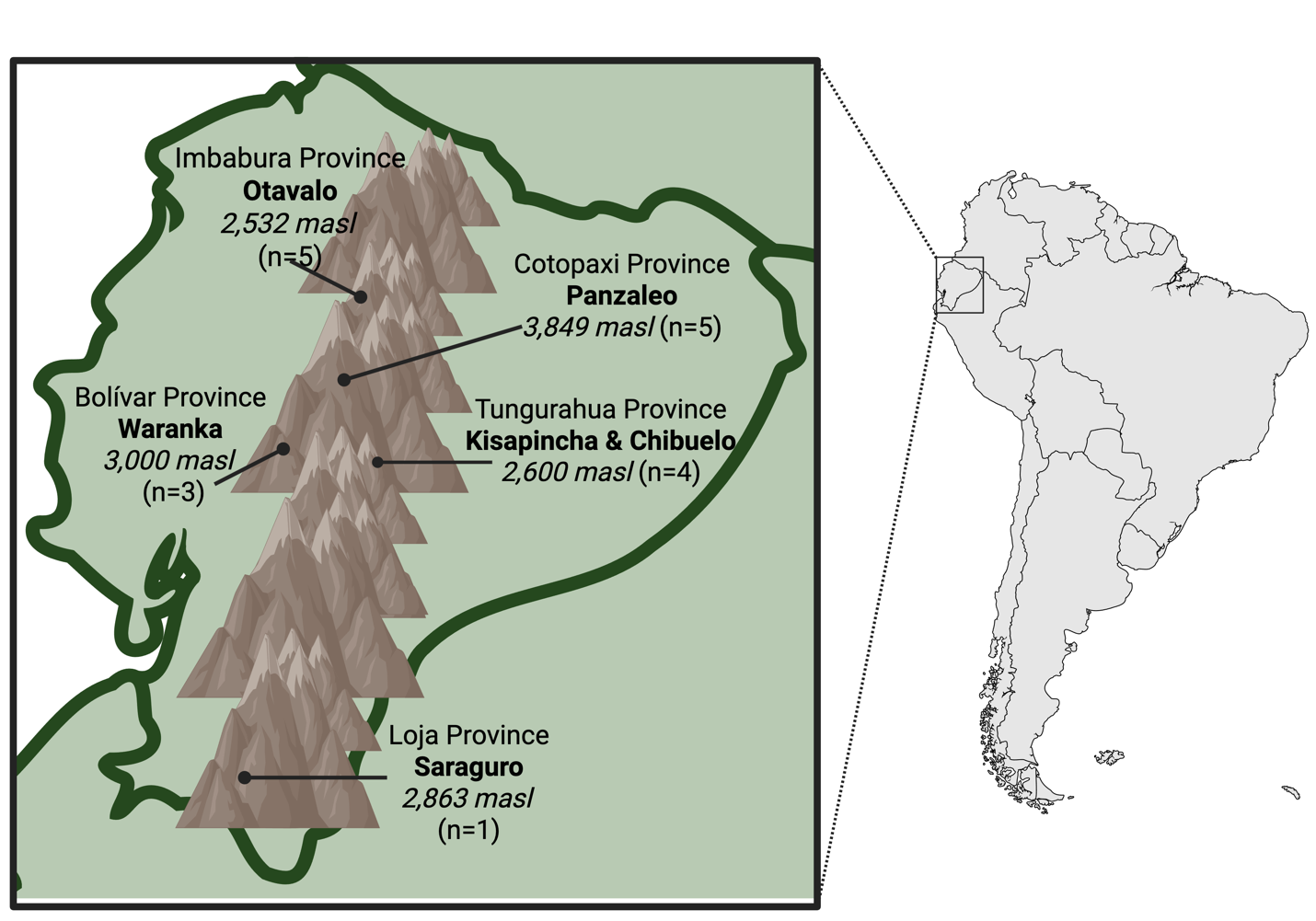
**

**Supplementary Figure 2A:** English translation of the informed consent form signed by all Kichwa volunteer donors.


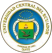


**Informed Consent Form**

**Project objective:** to determine the existing genetic relationships between the ethnic  population groups of Ecuador. Population genetics is the study of the forces that alter  the genetic makeup of a species. It deals with micro-evolutionary change  mechanisms: mutation, natural selection, gene flow, and gene drift.  **Voluntary participation:** You can freely choose whether or not you want to  participate in this study. There is no type of penalty if you do not wish to participate  or wish to withdraw from the study in any of this project's phases. We do not ask for  any explanation for your withdrawal. You must understand what is required of you in  this project, so we are always ready to answer your questions or clarify your doubts;  In addition to providing detailed information on the phase of the project where we  are, please ask the interviewer or contact one of the people named at the end of this  sheet. Your participation in this project has no cost.

**Blood collection**: A healthcare professional punctures one of your fingers and the  blood are collected on an FTA® Whatman card.

**Confidentiality**: All information you provide we kept entirely confidential, and we use  it only for research purposes without any connection to your name. We share the  general results of the research only with the scientific community in general.  **Risks and benefits:** Because the method is only to take a blood sample (conventional  method of diagnosis), there are no risks to your health. The benefit of their  participation is to be able to establish the mechanisms of micro-evolutionary change:  mutation, natural selection, gene flow, and gene drift. This research will help the  scientific community to understand the genetic relationships that exist among  modern Ecuadorians.

Name of person taking the sample:

Location:

Canton:

Province:


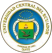


**Informed Consent**

I,____________________________________________________________ ID number______________________________________________________

I come freely, voluntarily, altruistically to donate a sample of my blood  obtained from one of my fingers, and that will be collected on an FTA card, for  subsequent DNA analysis for anthropological, population and research  purposes genetics genomics. The sample does NOT be used for diagnostic or  individual treatment purposes. The samples obtained are anonymous,  properly archived, and the study's genetic data are confidential. The donated  sample may be used for all types of DNA analysis for scientific research  purposes and may not be used for commercial or other purposes not  authorized in this consent. I also declare that I support scientific research as a  mechanism for social development and advancement of knowledge, so my  participation in this project is selfless and non-profit. I authorize the study  researchers to use the donated sample in the proposed research or others  derived from the knowledge obtained through it.

I declare that I have read this document, and I fully agree with it.  Sign:

Date:

**Supplementary Figure 2B:** Agreement signed with Ashaninka Indigenous community.

**Supplementary Figure 3:** Volcano plots visualizing sensitivity analysis comparing the percent of differential cytosine methylation (A) with males (1388 DMCs) and (B) without males (1455 DMCs) generated using Methylkit^S1^.


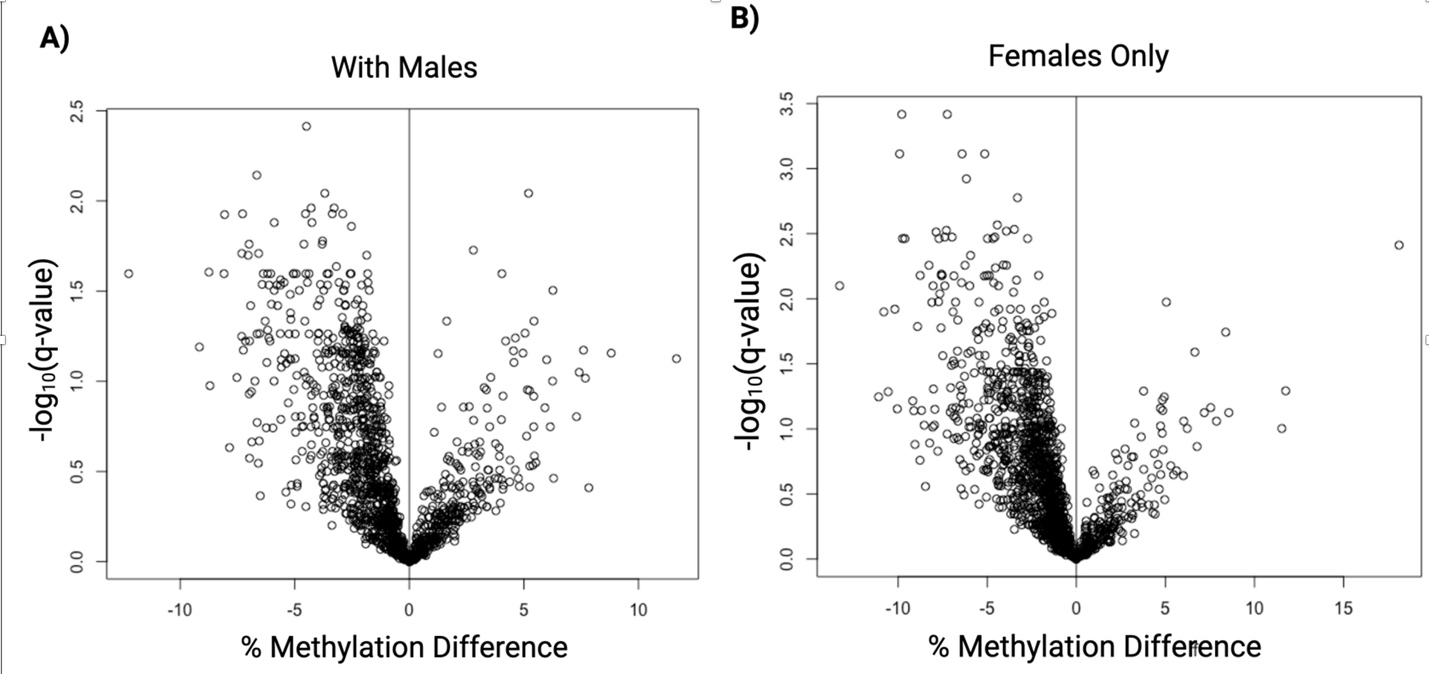


**Supplementary Figure 4:** Volcano plots of the DMRs calculated at minimum differential methylation thresholds of (A) 5%, (B) 10%, (C) 25%, and (D) 50% generated using Methylkit^S1^.
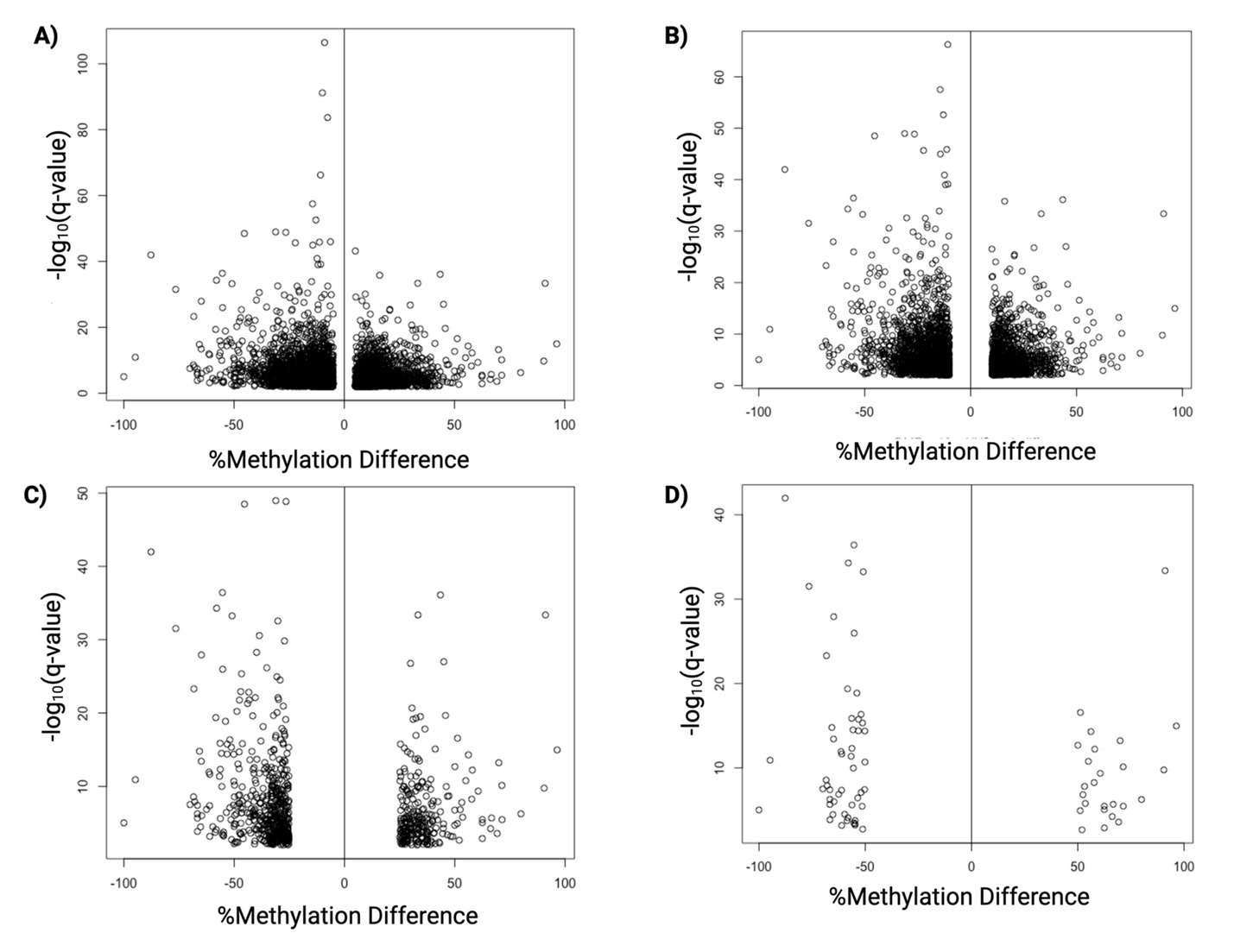


**
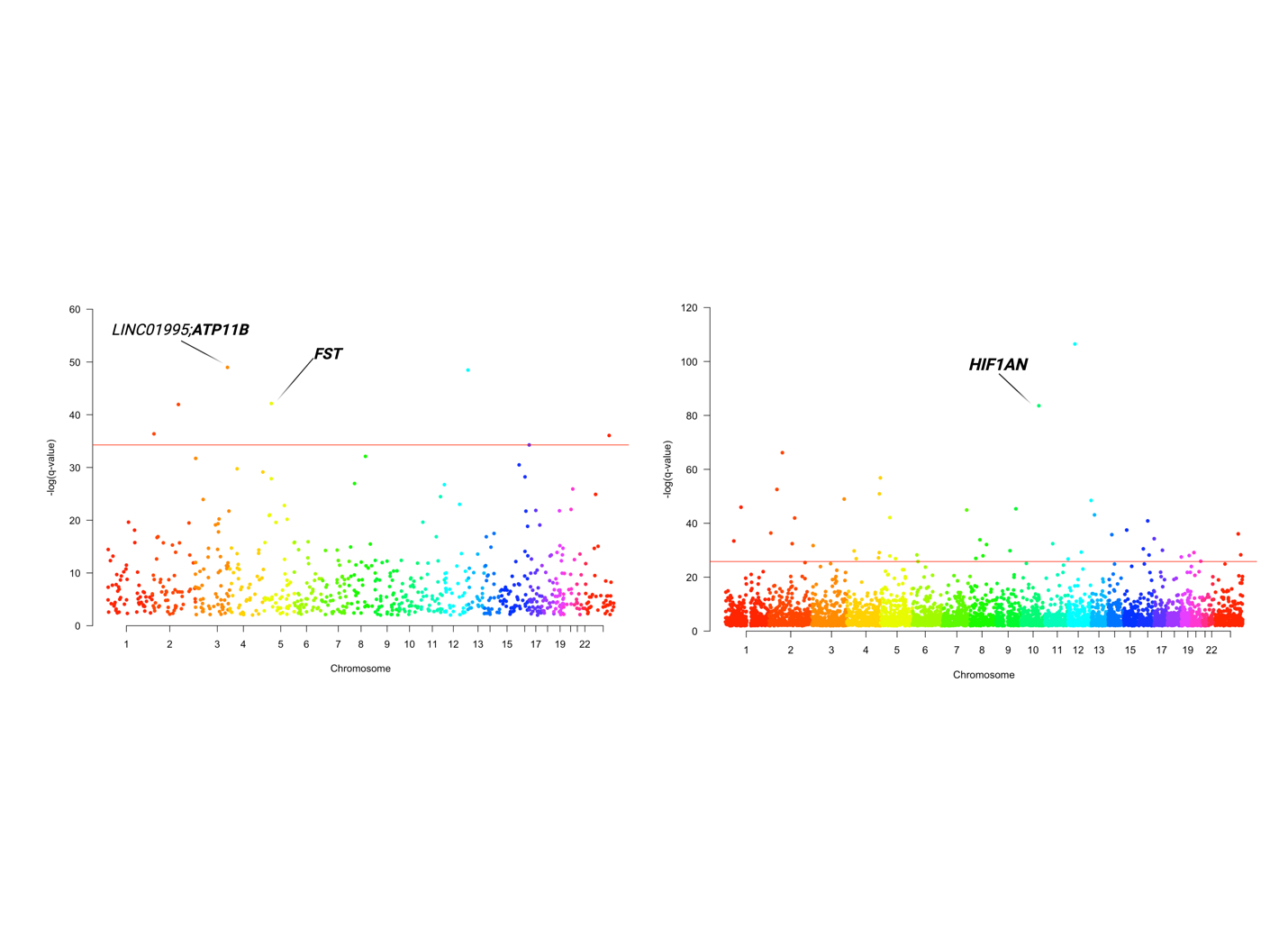
Supplementary Figure 5:** Manhattan plots of the DMRs calculated including the three admixted individuals at minimum differential methylation thresholds of (A) 5%, (B) 25% generated using Methylkit^S1^.


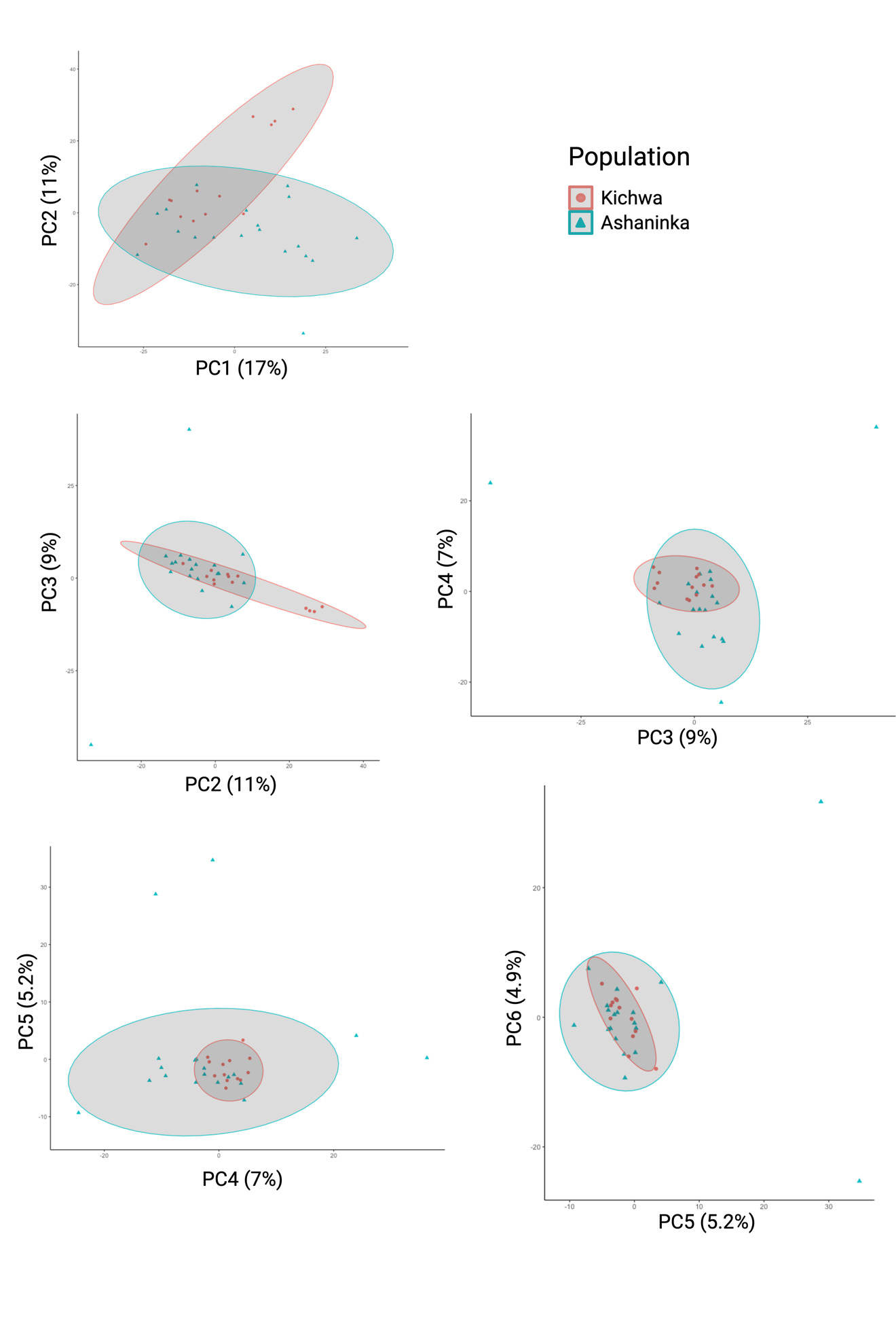
**Supplementary Figure 6:** PCA plots of PCs 1-6 from the DMC data generated using Methylkit^S1^.

**Supplementary Table 1:** The Sample IDs and population names of the individuals from the Human Genome Diversity Project^S2^ included in our analyses.

| **Sample ID** | | **Region** | |  | **Sample ID** | **Population** |
| --- | --- | --- | --- | --- | --- | --- |
| HGDP00449 | | Africa | |  | HGDP00704 | Americas |
| HGDP00450 | | Africa | |  | HGDP00706 | Americas |
| HGDP00456 | | Africa | |  | HGDP00708 | Americas |
| HGDP00462 | | Africa | |  | HGDP00710 | Americas |
| HGDP00463 | | Africa | |  | HGDP00970 | Americas |
| HGDP00467 | | Africa | |  | HGDP00995 | Americas |
| HGDP00471 | | Africa | |  | HGDP00998 | Americas |
| HGDP00474 | | Africa | |  | HGDP00999 | Americas |
| HGDP00476 | | Africa | |  | HGDP01001 | Americas |
| HGDP00478 | | Africa | |  | HGDP01009 | Americas |
| HGDP00982 | | Africa | |  | HGDP01010 | Americas |
| HGDP00984 | | Africa | |  | HGDP01012 | Americas |
| HGDP01149 | | Europe | |  | HGDP01013 | Americas |
| HGDP01151 | | Europe | |  | HGDP01014 | Americas |
| HGDP01152 | | Europe | |  | HGDP01015 | Americas |
| HGDP01153 | | Europe | |  | HGDP01018 | Americas |
| HGDP01155 | | Europe | |  | HGDP01019 | Americas |
| HGDP01156 | | Europe | |  | HGDP00854 | Americas |
| HGDP01157 | | Europe | |  | HGDP00855 | Americas |
| HGDP01171 | | Europe | |  | HGDP00856 | Americas |
| HGDP01172 | | Europe | |  | HGDP00706 | Americas |
| HGDP01173 | | Europe | |  | HGDP00708 | Americas |
| HGDP01174 | | Europe | |  | HGDP00710 | Americas |
| HGDP01177 | | Europe | |  | HGDP00970 | Americas |
| HGDP00776 | | East Asia | |  | HGDP00995 | Americas |
| HGDP00777 | | East Asia | |  | HGDP00998 | Americas |
| HGDP00780 | | East Asia | |  | HGDP00999 | Americas |
| HGDP00782 | | East Asia | |  | HGDP01001 | Americas |
| HGDP00811 | | East Asia | |  | HGDP01009 | Americas |
| HGDP00812 | | East Asia | |  | HGDP01010 | Americas |
| HGDP00813 | | East Asia | |  | HGDP01012 | Americas |
| HGDP00818 | | East Asia | |  | HGDP01013 | Americas |
| HGDP00821 | | East Asia | |  | HGDP01014 | Americas |
| HGDP00971 | | East Asia | |  | HGDP01015 | Americas |
| HGDP00972 | | East Asia | |  | HGDP01018 | Americas |
| HGDP00974 | | East Asia | |  | HGDP01019 | Americas |
| HGDP00975 | | East Asia | |  | HGDP00854 | Americas |
| HGDP00976 | | East Asia | |  |  |  |
| HGDP00702 | | Americas | |  |  |  |
| HGDP00703 | | Americas | |  |  |  |
| **Sample ID** | **Population** | |  |  |  |  |
| HGDP00855 | Americas | |  |  |  |  |
| HGDP00856 | Americas | |  |  |  |  |
| HGDP01041 | Americas | |  |  |  |  |
| HGDP01043 | Americas | |  |  |  |  |
| HGDP01044 | Americas | |  |  |  |  |
| HGDP01047 | Americas | |  |  |  |  |
| HGDP01050 | Americas | |  |  |  |  |
| HGDP01053 | Americas | |  |  |  |  |
| HGDP01055 | Americas | |  |  |  |  |
| HGDP01056 | Americas | |  |  |  |  |
| HGDP01057 | Americas | |  |  |  |  |
| HGDP01058 | Americas | |  |  |  |  |
| HGDP01059 | Americas | |  |  |  |  |
| HGDP01060 | Americas | |  |  |  |  |
| HGDP00832 | Americas | |  |  |  |  |
| HGDP00837 | Americas | |  |  |  |  |
| HGDP00838 | Americas | |  |  |  |  |
| HGDP00843 | Americas | |  |  |  |  |
| HGDP00845 | Americas | |  |  |  |  |
| HGDP00846 | Americas | |  |  |  |  |
| HGDP00849 | Americas | |  |  |  |  |
| HGDP00852 | Americas | |  |  |  |  |

**Supplementary Figure 7:** Major panels of the poster that will be presented to the community. This will be available in English and Spanish.


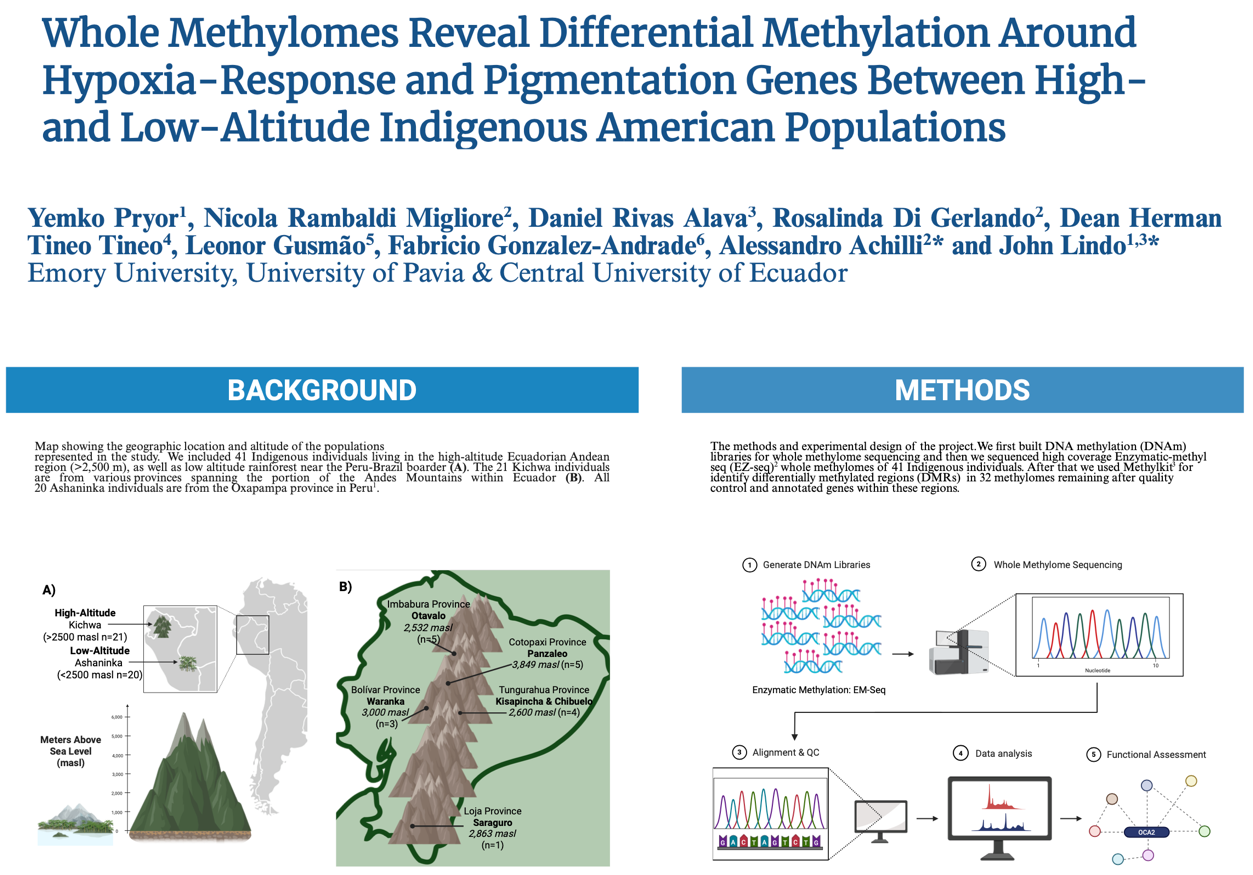


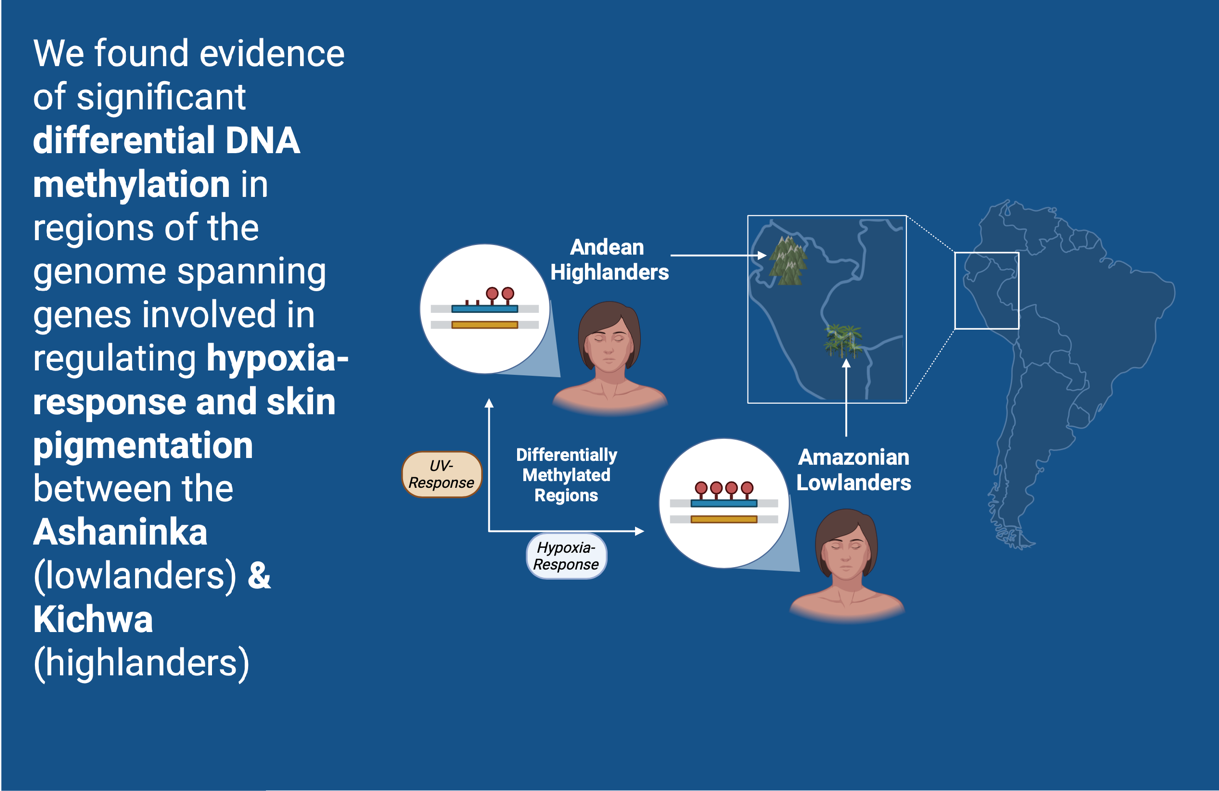


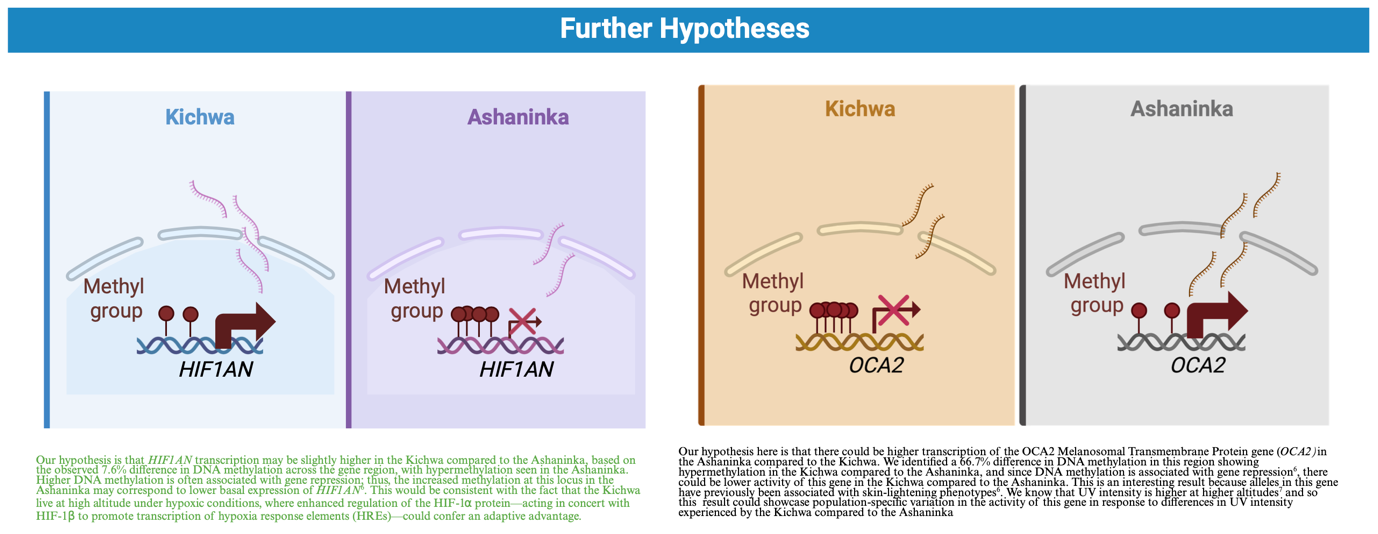
**
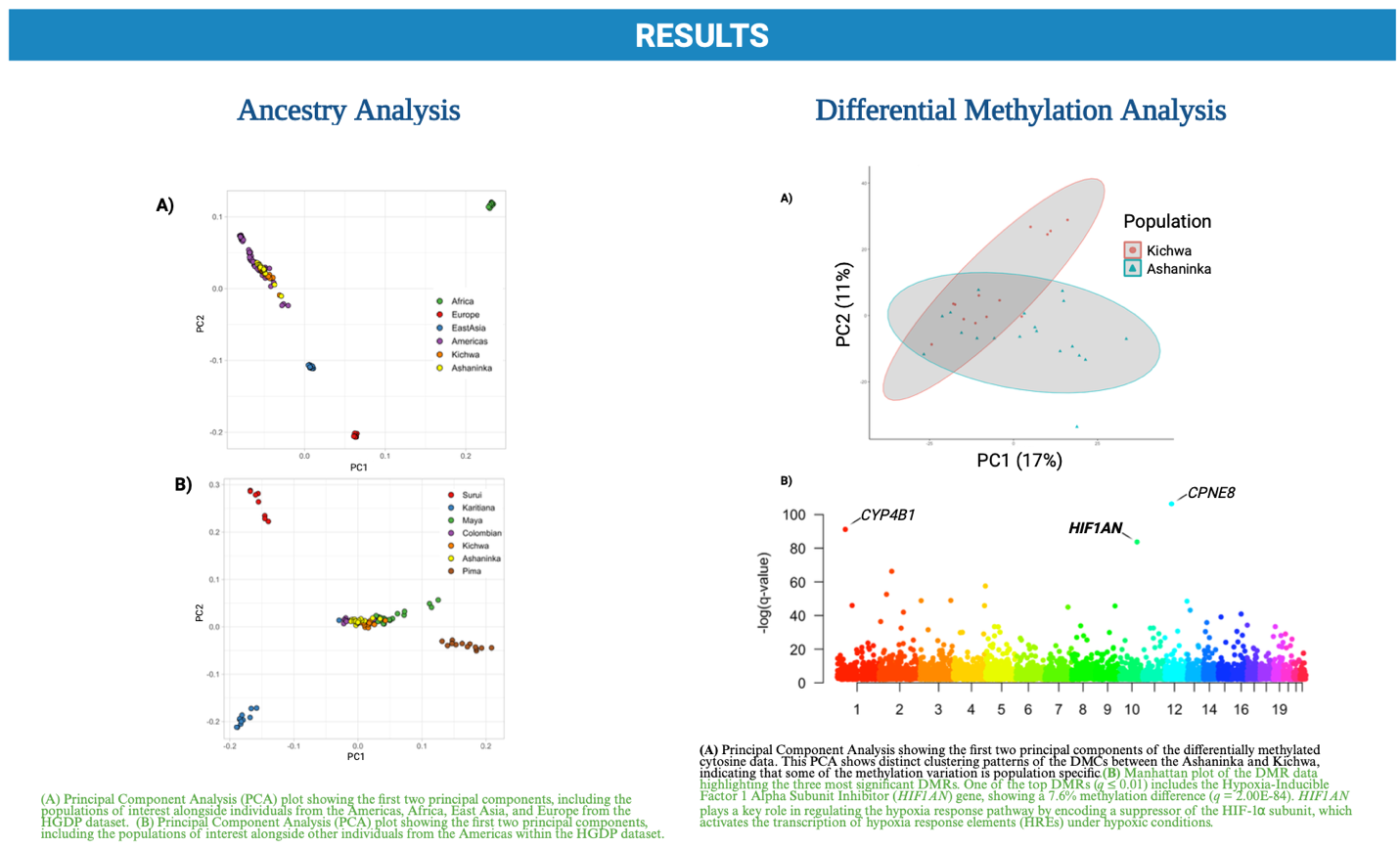
**


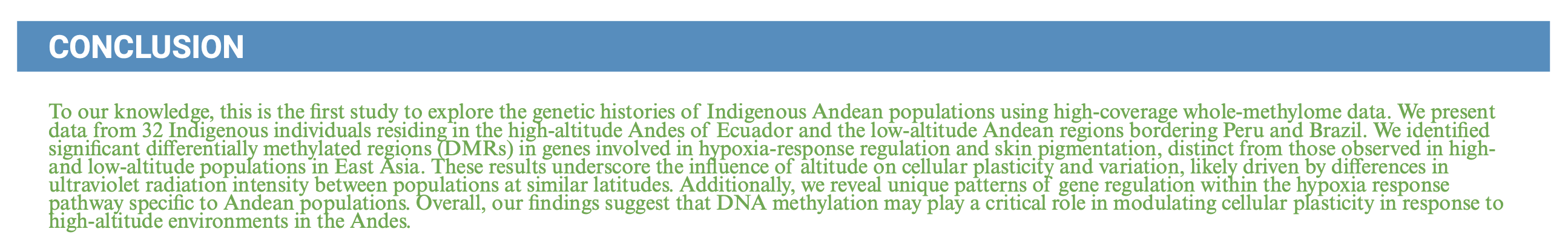


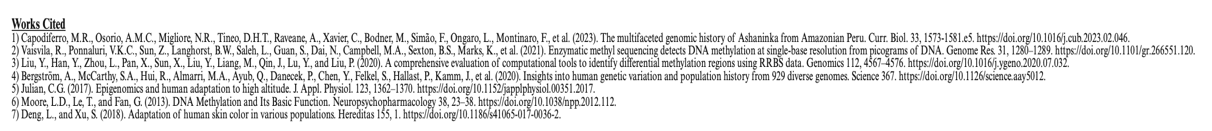


**Supplementary Table 2:** The sample IDs, altitude, in meters above sea level (masl). Bolded sample IDs indicate those included in the final differential methylation analysis.

| **SAMPLE ID** | **ALTITUDE** | **MEAN METHYL-SEQ COVERAGE** |
| --- | --- | --- |
| K-Chib-2A | 2,580 masl | 23.53681818 |
| K-Chib-7A | 2,580 masl | 25.51954545 |
| **K-Kish-1A** | 2,600 masl | 12.41 |
| **K-Kish-2A** | 2,600 masl | 19.35045455 |
| K-Kish-5A | 2,600 masl | 14.75772727 |
| **K-Otau-2A** | 2,532 masl | 9.740454545 |
| **K-Otau-7A** | 2,532 masl | 9.138636364 |
| **K-Otau-7B** | 2,532 masl | 14.17772727 |
| **K-Otau-8A** | 2,532 masl | 11.87 |
| **K-Otau-9B** | 2,532 masl | 6.536818182 |
| **K-Panza-1A** | 3,849 masl | 6.489545455 |
| **K-Panza-1B** | 3,849 masl | 5.960909091 |
| **K-Panza-2A** | 3,849 masl | 9.751818182 |
| K-Panza-4B | 3,849 masl | 14.67863636 |
| **K-Panza-7A** | 3,849 masl | 5.207619048 |
| K-Panza-10B | 3,849 masl | 4.383636364 |
| **K-Sara-10B** | 2,863 masl | 4.688636364 |
| K-Wara-5B | 3,000 masl | 7.245454545 |
| K-Wara-7A | 3,000 masl | 6.191818182 |
| **K-Wara-7B** | 3,000 masl | 7.527727273 |
| **AD 021** | 250 masl | 4.001363636 |
| **AD 032** | 250 masl | 6.115909091 |
| **AD 038** | 250 masl | 4.846363636 |
| **AD 045** | 250 masl | 7.306363636 |
| **AD 049** | 250 masl | 3.724545455 |
| **AD 057** | 250 masl | 4.677727273 |
| **AD 060** | 250 masl | 4.354090909 |
| **AD 073** | 250 masl | 4.772727273 |
| **AD 093** | 250 masl | 4.550909091 |
| **AD 095** | 250 masl | 3.67 |
| **AD 098** | 250 masl | 2.455 |
| **AD 124** | 250 masl | 3.419090909 |
| **AD 128** | 250 masl | 2.982727273 |
| **AD 137** | 250 masl | 2.467727273 |
| **AD 139** | 250 masl | 1.483181818 |
| **AD 143** | 250 masl | 2.499545455 |
| **AD 150** | 250 masl | 2.266818182 |
| **AD 164** | 250 masl | 3.272727273 |
| **AD 179** | 250 masl | 3.293181818 |

**Supplementary Table 3: Top 15 of FDR and DM Significant DMRs at 25% Differential Methylation Threshold**

Top 15 of FDR Significant DMRs at 25% Differential

| **CHR** | **Start** | **End** | **Region** | **Gene** | **Methylkit pvalue** | **Methylkit qvalue** | **Methylation Difference** |
| --- | --- | --- | --- | --- | --- | --- | --- |
| Chr 3 | 182384184 | 182384423 | intergenic | LINC01995(dist=159272),ATP11B(dist=126869) | 1.52E-52 | 8.25E-50 | -31.124697 |
| Chr 12 | 131199824 | 131200157 | intronic | RIMBP2 | 5.47E-52 | 2.73E-49 | -45.318215 |
| Chr 2 | 144694330 | 144695180 | ncRNA_exonic | LOC101928386 | 3.27E-45 | 9.65E-43 | -87.700275 |
| Chr 2 | 9606307 | 9606563 | intronic | CPSF3 | 1.41E-39 | 3.41E-37 | -55.317674 |
| Chr X | 130251619 | 130251893 | intergenic | ARHGAP36(dist=27760),IGSF1(dist=155590) | 3.03E-39 | 7.03E-37 | 43.5101353 |
| Chr17 | 617755 | 618928 | exonic | VPS53 | 2.13E-37 | 4.62E-35 | -57.968078 |
| Chr5 | 52777788 | 52777996 | intronic | FST | 2.01E-36 | 3.96E-34 | 33.340731 |
| Chr5 | 75037353 | 75037569 | intergenic | LOC441087(dist=10558),SV2C(dist=341639) | 2.80E-36 | 5.34E-34 | -50.944242 |
| Chr11 | 65585389 | 65585884 | intergenic | OVOL1(dist=20704),SNX32(dist=15550) | 1.42E-35 | 2.63E-33 | -30.18126 |
| Chr5 | 124788081 | 124788359 | intergenic | LOC101927421(dist=84654),LINC02240(dist=40595) | 1.35E-34 | 2.25E-32 | -42.945406 |
| Chr19 | 12273794 | 12274291 | exonic | ZNF136 | 1.38E-34 | 2.25E-32 | 87.1287129 |
| Chr3 | 48129925 | 48130903 | UTR5 | MAP4 | 1.66E-34 | 2.63E-32 | -76.470588 |
| Chr16 | 28833904 | 28835873 | exonic | ATXN2L | 1.65E-33 | 2.43E-31 | -38.571708 |
| Chr4 | 41050823 | 41051593 | intronic | APBB2 | 1.00E-32 | 1.30E-30 | -27.204456 |
| Chr5 | 52705488 | 52705769 | intergenic | LOC257396(dist=294532),FST(dist=70690) | 6.30E-32 | 7.87E-30 | -64.848485 |

**Supplementary Table 4: Top 1% of FDR Significant DMRs at 5% Differential Methylation Threshold**

Top 1% of FDR Significant DMRs at 5% Differential Methylation Threshold.

| **CHR​** | **Start​** | **End​** | **Region​** | **Gene​** | **Methylkit ​**  **pvalue​** | **Methlykit ​**  **qvalue​** | **Methylation Difference​** |
| --- | --- | --- | --- | --- | --- | --- | --- |
| Chr12​ | 39538383​ | 39539500​ | intergenic​ | *CPNE8(dist=238990),​KIF21A(dist=147530)​* | 5.18E-111​ | 1.68E-107​ | -8.9242796​ |
| Chr1​ | 47239608​ | 47239889​ | intergenic​ | *EFCAB14(dist=54822),CYP4B1(dist=24829)​* | 1.97E-95​ | 4.27E-92​ | -9.9132891​ |
| Chr10​ | 102295548​ | 102295910​ | exonic​ | *HIF1AN​* | 1.24E-87​ | 1.61E-84​ | -7.5642837​ |
| Chr3​ | 6532266​ | 6532599​ | intergenic​ | *MIR4790(dist=1240326),GRM7-AS3(dist=141446)​* | 7.25E-85​ | 7.86E-82​ | -24.580196​ |
| Chr2​ | 75146923​ | 75147560​ | ncRNA_intronic​ | *LINC01291​* | 4.23E-70​ | 3.93E-67​ | -10.770373​ |
| chr2​ | 44314288​ | 44314564​ | intergenic​ | *LRPPRC(dist=91160),PPM1B(dist=81378)​* | 3.16E-56​ | 2.55E-53​ | -12.87287​ |
| chr3​ | 182384184​ | 182384423​ | intergenic​ | *LINC01995(dist=159272),ATP11B(dist=126869)​* | 1.52E-52​ | 1.09E-49​ | -31.124697​ |
| chr3​ | 6532266​ | 6532599​ | intergenic​ | *MIR4790(dist=1240326),GRM7-AS3(dist=141446)​* | 2.21E-52​ | 1.43E-49​ | -26.513109​ |
| chr12​ | 131199824​ | 131200157​ | intronic​ | *RIMBP2​* | 5.47E-52​ | 3.21E-49​ | -45.318215​ |
| chr1​ | 87738595​ | 87738876​ | intergenic​ | *LINC02801(dist=21581),LMO4(dist=55687)​* | 1.95E-49​ | 1.05E-46​ | -6.2413362​ |
| chr4​ | 184319564​ | 184320198​ | intergenic​ | *CLDN24(dist=75985),CDKN2AIP(dist=45591)​* | 2.76E-49​ | 1.37E-46​ | -11.263182​ |
| chr9​ | 112360875​ | 112361110​ | intergenic​ | *MIR3927(dist=87050),PALM2AKAP2(dist=41958)​* | 4.68E-49​ | 2.16E-46​ | -22.214188​ |
| chr7​ | 137686291​ | 137687056​ | exonic​ | *CREB3L2​* | 2.47E-48​ | 1.06E-45​ | -14.278097​ |
| chr13​ | 36049570​ | 36050159​ | exonic​ | *MAB21L1​* | 1.74E-46​ | 6.61E-44​ | 5.0166362​ |
| chr2​ | 144694330​ | 144695180​ | ncRNA_exonic​ | *LOC101928386​* | 3.27E-45​ | 1.05E-42​ | -87.700275​ |
| chr16​ | 53737617​ | 53738235​ | exonic​ | *FTO​* | 4.11E-44​ | 1.26E-41​ | -12.33516​ |
| chr15​ | 37172473​ | 37173018​ | ncRNA_intronic​ | *LOC145845​* | 2.52E-42​ | 7.38E-40​ | -10.67874​ |
| chrX​ | 11129276​ | 11129758​ | UTR5​ | *HCCS​* | 3.51E-42​ | 9.84E-40​ | -11.867869​ |
| chr2​ | 9606307​ | 9606563​ | intronic​ | *CPSF3​* | 1.41E-39​ | 3.81E-37​ | -55.317674​ |
| chrX​ | 130251619​ | 130251893​ | intergenic​ | *ARHGAP36(dist=27760),IGSF1(dist=155590)​* | 3.03E-39​ | 7.83E-37​ | 43.5101353​ |
| chr14​ | 39582041​ | 39582318​ | intergenic​ | *SEC23A-AS1(dist=9025),GEMIN2(dist=1209)​* | 6.22E-39​ | 1.54E-36​ | 16.0634052​ |
| chr17​ | 617755​ | 618928​ | exonic​ | *VPS53​* | 2.13E-37​ | 5.10E-35​ | -57.968078​ |
| chr8​ | 54507107​ | 54507358​ | intergenic​ | *LOC100507516(dist=70582),ATP6V1H(dist=120745)​* | 5.67E-37​ | 1.26E-34​ | -14.809984​ |
| chr5​ | 52777788​ | 52777996​ | intronic​ | *FST​* | 2.01E-36​ | 4.21E-34​ | 33.340731​ |
| chr19​ | 12273794​ | 12274291​ | exonic​ | *ZNF136​* | 2.02E-36​ | 4.21E-34​ | 91.1111111​ |
| chr5​ | 75037353​ | 75037569​ | intergenic​ | *LOC441087(dist=10558),SV2C(dist=341639)​* | 2.80E-36​ | 5.64E-34​ | -50.944242​ |
| chr11​ | 65585389​ | 65585884​ | intergenic​ | *OVOL1(dist=20704),SNX32(dist=15550)​* | 1.42E-35​ | 2.77E-33​ | -30.18126​ |
| chr2​ | 130737205​ | 130737718​ | exonic​ | *RAB6C​* | 1.70E-35​ | 3.20E-33​ | -21.392745​ |
| chr11​ | 46959142​ | 46959384​ | intronic​ | *C11orf49​* | 1.73E-35​ | 3.20E-33​ | -7.5040212​ |
| chr3​ | 48129925​ | 48130903​ | UTR5​ | *MAP4​* | 1.66E-34​ | 2.97E-32​ | -76.470588​ |
| chrX​ | 144435385​ | 144435670​ | intergenic​ | *SPANXN1(dist=97657),SLITRK2(dist=463677)​* | 3.48E-34​ | 6.08E-32​ | -20.496776​ |
| chr12​ | 75784799​ | 75785241​ | exonic​ | *GLIPR1L2​* | 1.19E-33​ | 2.02E-31​ | -20.452852​ |
| chr16​ | 28833904​ | 28835873​ | exonic​ | *ATXN2L​* | 1.65E-33​ | 2.73E-31​ | -38.571708​ |
| chr14​ | 31683281​ | 31683555​ | intergenic​ | *HECTD1(dist=6282),HEATR5A(dist=77439)​* | 2.35E-33​ | 3.79E-31​ | -16.625797​ |
| chr5​ | 85063388​ | 85063616​ | intergenic​ | *EDIL3(dist=1382690),NBPF22P(dist=514646)​* | 4.80E-33​ | 7.56E-31​ | 9.51019704​ |
| chr4​ | 52942978​ | 52943248​ | exonic​ | *SPATA18​* | 6.80E-33​ | 1.05E-30​ | -9.0220151​ |
| chr9​ | 79073160​ | 79073394​ | intronic​ | *GCNT1​* | 8.11E-33​ | 1.22E-30​ | -5.9035069​ |
| chr4​ | 41050823​ | 41051593​ | intronic​ | *APBB2​* | 1.00E-32​ | 1.47E-30​ | -27.204456​ |
| chr11​ | 97784689​ | 97784962​ | intergenic​ | *LINC02737(dist=1536775),CNTN5(dist=1106717)​* | 4.51E-32​ | 6.47E-30​ | 5.27041076​ |
| chr20​ | 13765166​ | 13766017​ | exonic​ | *NDUFAF5​* | 6.65E-32​ | 9.34E-30​ | -24.675818​ |
| chr4​ | 179111747​ | 179112022​ | intergenic​ | *LINC01098(dist=199843),NONE(dist=NONE)​* | 6.87E-32​ | 9.44E-30​ | -10.30835​ |
| chr16​ | 61328400​ | 61328675​ | intergenic​ | *MIR4426(dist=238730),CDH8(dist=352480)​* | 3.99E-31​ | 5.37E-29​ | -39.827248​ |
| chr19​ | 45999761​ | 46002686​ | exonic​ | *PPM1N,RTN2​* | 6.26E-31​ | 8.25E-29​ | 8.04161162​ |
| chr8​ | 71933759​ | 71934032​ | intergenic​ | *XKR9(dist=285582),EYA1(dist=175636)​* | 8.06E-31​ | 1.04E-28​ | -22.294791​ |
| chr5​ | 52705488​ | 52705769​ | intergenic​ | *LOC257396(dist=294532),FST(dist=70690)​* | 9.42E-31​ | 1.19E-28​ | -64.848485​ |
| chr19​ | 1226490​ | 1251179​ | exonic​ | *ATP5F1D,CBARP,MIDN,STK11​* | 2.27E-30​ | 2.82E-28​ | -18.28094​ |
| chr8​ | 31318062​ | 31318341​ | intergenic​ | *WRN(dist=284409),NRG1(dist=178927)​* | 8.25E-30​ | 1.01E-27​ | 45.0230415​ |
| chr14​ | 54955388​ | 54955831​ | exonic​ | *GMFB​* | 1.23E-29​ | 1.44E-27​ | -11.832404​ |
| chr12​ | 718174​ | 718561​ | intronic​ | *NINJ2​* | 1.48E-29​ | 1.71E-27​ | 29.9257026​ |
| chr6​ | 26146122​ | 26146396​ | intergenic​ | *H2AC6(dist=21204),H1-4(dist=10161)​* | 2.79E-29​ | 3.10E-27​ | -6.4034447​ |

**Supplementary Figure 8: Batch Effect Estimation**

PCA ploting the first calculated using *assocComp*, looking at the correlation between the DMC data and batch effects^S1^.


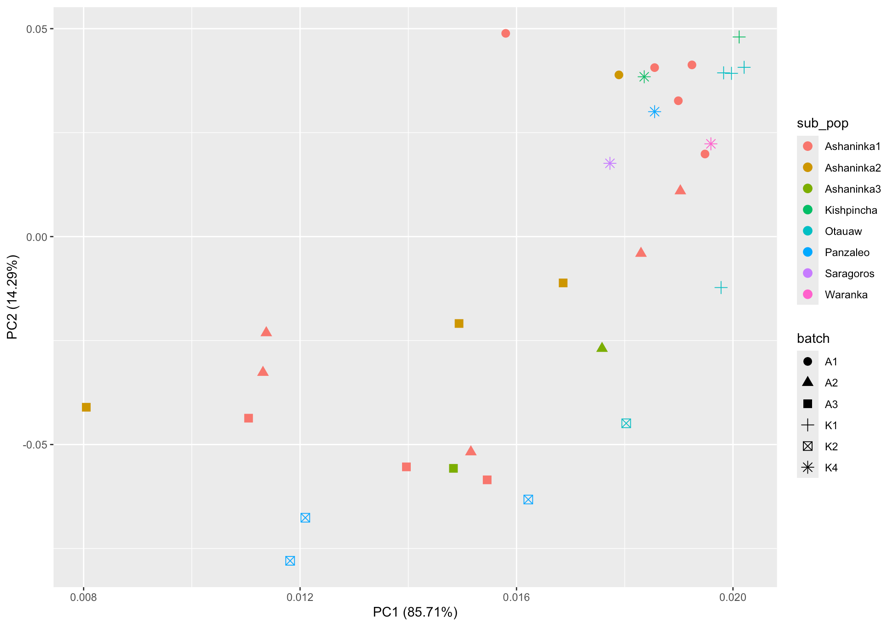


**Supplementary Figure 9: DMRs After Age Estimation**

Volcano Plots comparing the DMRs at (A) 5%, (B) 10%, (C) 25%, and (50%) differential methylation thresholds before and after adding age as a covariate. Age was estimated using *methylclock*^S3^.


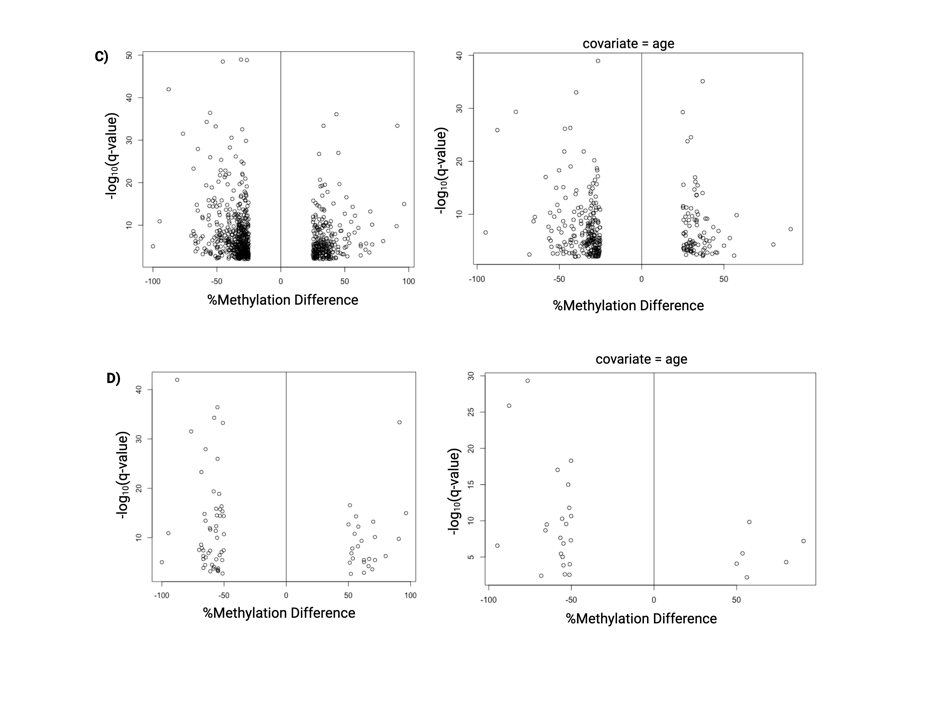

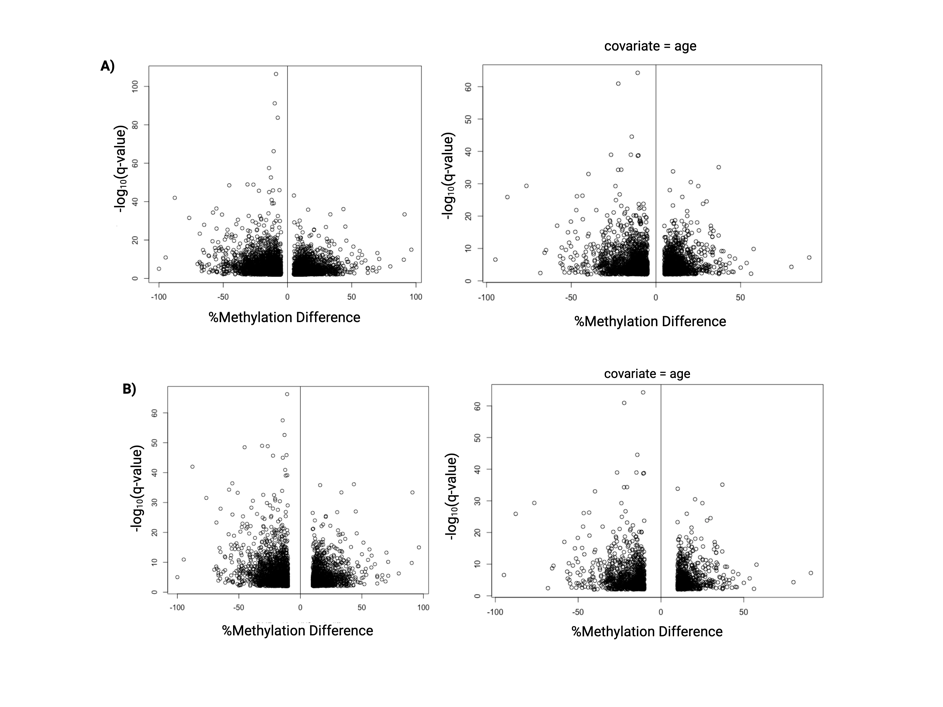


**Supplementary Figure 10: Cellular Composition Estimation**

A heat map showing the cell-type proportion estimations within our data, after attempting to map our whole-methylome CpG data to the array-based Infinium Methylation EPIC v1.0 B5 Manifest data probe IDs, using the Comprehensive Human Methylome Atlas^S4^ as a reference within *deconvR*^S5^*.*


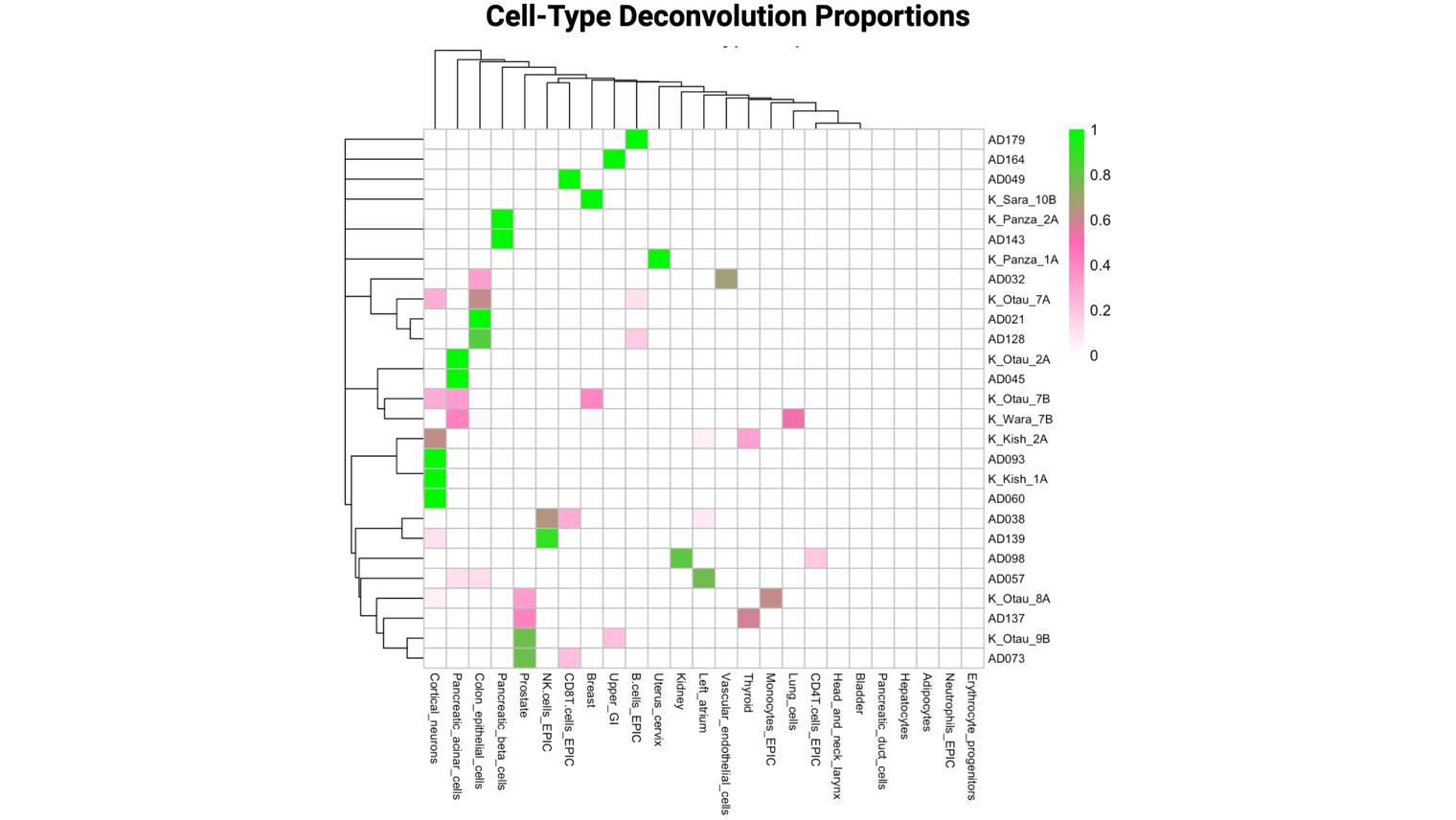


**Supplementary Table 5: Hypoxia Response Pathway Genes within FDR Significant DMRs.** Negative methylation differences indicate hypermethylation in the Ashaninka, while positive values indicate hypermethylation in the Kichwa. Pathways identified with Reactome^S6^.

| **Chromosome**​ | **Start**​ | **End**​ | **Gene**​ | **MethylKit**​  **pvalue**​ | **MethylKit**​  **qvalue**​ | **Methylation Difference**​ | **Reactome Pathway**​ |
| --- | --- | --- | --- | --- | --- | --- | --- |
| Chr 10​ | 102295548​ | 102295910​ | *HIF1AN*​ | 1.24E-87​ | 2.00E-84​ | -7.5642837​ | Cellular Response to Hypoxia​ |
| Chr 12​ | 122326387​ | 122327044​ | *PSMD9*​ | 8.48E-15​ | 1.66E-13​ | 13.740458​ | Cellular Response to Hypoxia​ |
| Chr 22​ | 41460014​ | 41460272​ | *RBX1*(dist=90701)​ | 9.87E-14​ | 1.61E-12​ | -38.623207​ | Cellular Response to Hypoxia​ |
| Chr 6​ | 170862124​ | 170862480​ | *PSMB1*​ | 4.74E-10​ | 3.70E-09​ | -11.216808​ | Cellular Response to Hypoxia​ |
| Chr 6​ | 139717915​ | 139718325​ | *CITED2*(dist=22130)​ | 2.04E-07​ | 9.20E-07​ | 18.0722892​ | Cellular Response to Hypoxia​ |
| Chr 14​ | 23496316​ | 23496547​ | *PSMB5*​ | 3.21E-07​ | 1.39E-06​ | 23.3918129​ | Cellular Response to Hypoxia​ |
| Chr 2​ | 162270888​ | 162271413​ | *PSMD14*(dist=2660)​ | 5.10E-07​ | 2.08E-06​ | 6.42019626​ | Cellular Response to Hypoxia​ |
| Chr 14​ | 90678771​ | 90678998​ | *PSMC1*(dist=43899)​ | 6.11E-07​ | 2.45E-06​ | -11.236568​ | Cellular Response to Hypoxia​ |
| Chr 18​ | 23777436​ | 23777673​ | *PSMA8*(dist=4117)​ | 1.47E-06​ | 5.27E-06​ | -40.602234​ | Cellular Response to Hypoxia​ |
| Chr 9​ | 127177366​ | 127177818​ | *PSMB7*​ | 1.98E-05​ | 5.21E-05​ | -17.619048​ | Cellular Response to Hypoxia​ |
| Chr 7​ | 96409459​ | 96409677​ | *SEM1*​ | 0.00026602​ | 0.00050335​ | 10.1333333​ | Cellular Response to Hypoxia​ |
| Chr 8​ | 74807047​ | 74807300​ | *ELOC*(dist=50071)​ | 0.00043624​ | 0.00077374​ | -8.8294379​ | Cellular Response to Hypoxia​ |
| Chr 5​ | 139016993​ | 139017668​ | *UBE2D2*​ | 0.00118542​ | 0.00185449​ | 15.3846154​ | Cellular Response to Hypoxia​ |
| Chr 1​ | 151226665​ | 151227238​ | *PSMD4*​ | 0.00163305​ | 0.00243376​ | 10.0664452​ | Cellular Response to Hypoxia​ |
| Chr 14​ | 34442323​ | 34442612​ | *EGLN3*​ | 0.00185159​ | 0.00270653​ | 9.4224924​ | Cellular Response to Hypoxia​ |
| Chr 20​ | 1094357​ | 1094642​ | *PSMF1*​ | 0.00334455​ | 0.00448324​ | 11.8055556​ | Cellular Response to Hypoxia​ |
| Chr 10​ | 35271325​ | 35271599​ | *CUL2*(dist=25880)​ | 0.00416922​ | 0.00539137​ | 5.28169014​ | Cellular Response to Hypoxia​ |
| Chr 6​ | 32820849​ | 32822370​ | *PSMB9,TAP1*​ | 0.00446742​ | 0.00571293​ | -15.52795​ | Cellular Response to Hypoxia​ |
| Chr 16​ | 3974407​ | 3974632​ | *CREBBP*(dist=43693)​ | 0.00685644​ | 0.00822407​ | -18.181818​ | Cellular Response to Hypoxia​ |

**Supplementary Table 6: Pigmentation-Associated Genes within FDR Significant DMRs**.

Negative methylation differences indicate hypermethylation in the Ashaninka, while positive values indicate hypermethylation in the Kichwa. Pathways identified with Reactome^S6^.

| **CHR** | **Start**​ | **End**​ | **Gene**​ | **Methylkit pvalue**​ | **Methylkit qvalue**​ | **Methylation Difference**​ | **Reactome Pathway**​ |
| --- | --- | --- | --- | --- | --- | --- | --- |
| Chr X​ | 77187083​ | 77188295​ | *ATP7A​* | 1.68E-14​ | 3.13E-13​ | 17.41573034​ | Ion influx/efflux at host-pathogen interface​ |
| Chr 19​ | 2143803​ | 2144042​ | *AP3D1​* | 2.68E-13​ | 4.01E-12​ | 22.42536889​ | N/A​ |
| Chr 9​ | 89226598​ | 89226957​ | *GAS1(dist=332320)​* | 9.40E-12​ | 1.03E-10​ | -32.63301011​ | Signal Transduction​ |
| Chr 12​ | 43618087​ | 43618442​ | *ADAMTS20(dist=129227)​* | 3.32E-11​ | 3.25E-10​ | -24.94703222​ | O-glycosylation of TSR domain-containing proteins​ |
| Chr 6​ | 153494724​ | 153494961​ | *OPRM1(dist=836670)​* | 6.22E-11​ | 5.83E-10​ | -10.06617547​ | Opioid Signaling​ |
| Chr 10​ | 123489217​ | 123489495​ | *FGFR2(dist=131245)​* | 9.93E-11​ | 8.96E-10​ | -32.97571993​ | PIP3 activates AKT signaling​ |
| Chr 4​ | 55888773​ | 55889003​ | *KIT​* | 2.92E-10​ | 2.38E-09​ | 8.131424912​ | Constitutive Signaling by Aberrant PI3K in Cancer​ |
| Chr 18​ | 2711502​ | 2711776​ | *SMCHD1​* | 6.23E-09​ | 3.97E-08​ | -9.304072756​ | N/A​ |
| Chr 19​ | 3093393​ | 3095055​ | *GNA11​* | 8.76E-08​ | 4.34E-07​ | -10.17521805​ | Signal Transduction​ |
| Chr 9​ | 12587748​ | 12587985​ | *TYRP1(dist=105400)​* | 1.68E-07​ | 7.74E-07​ | 14​ | Melanin Biosynthesis​ |
| Chr 10​ | 96337539​ | 96337823​ | *HELLS​* | 2.04E-07​ | 9.21E-07​ | -8.491302707​ | TGFBR3 expression​ |
| Chr 11​ | 88549102​ | 88549383​ | *GRM5​* | 2.18E-07​ | 9.74E-07​ | -18.32812771​ | GPCR downstream signaling​ |
| Chr 5​ | 77590137​ | 77590721​ | *AP3B1​* | 2.83E-07​ | 1.23E-06​ | -28.6194699​ | Golgi Associated Vesicle Biogenesis​ |
| Chr 7​ | 55000876​ | 55001135​ | *EGFR(dist=85575)​* | 4.25E-07​ | 1.77E-06​ | -26.11711449​ | PTK6 promotes HIF1A stabilization​ |
| Chr 15​ | 28147925​ | 28148465​ | *OCA2​* | 4.99E-07​ | 2.04E-06​ | 66.66666667​ | Melanin Biosynthesis​ |
| Chr 1​ | 28706589​ | 28706863​ | *PHACTR4​* | 1.75E-06​ | 6.16E-06​ | -24.21150278​ | N/A​ |
| Chr 12​ | 88828713​ | 88828949​ | *KITLG(dist=57621)​* | 2.61E-06​ | 8.80E-06​ | -11.02817974​ | Regulation of KIT signaling​ |
| Chr 11​ | 85917754​ | 85918020​ | *EED(dist=37406)​* | 1.20E-05​ | 3.35E-05​ | 14.31455004​ | Chromatin organization​ |
| Chr 11​ | 88906705​ | 88906983​ | *GRM5(dist=107555),​*  *TYR(dist=4060)​* | 4.80E-05​ | 0.000113717​ | 11.5479791​ | GPCR downstream signaling; Melanin Biosynthesis​ |
| Chr 16​ | 90003618​ | 90003845​ | *TUBB3(dist=1113),​*  *DEF8(dist=11294)​* | 0.000101661​ | 0.000219279​ | 17.64705882​ | Microtubule-dependent trafficking of connexons from Golgi to the plasma membrane​ |
| Chr 3​ | 158551099​ | 158551377​ | *MFSD1(dist=3591)​* | 0.000133719​ | 0.000278924​ | -26.53061224​ | GPCR downstream signaling​ |
| Chr 6​ | 56498046​ | 56498362​ | *DST​* | 0.000158031​ | 0.000322653​ | -47.16981132​ | Assembly of collagen fibrils and other multimeric structures​ |
| Chr 10​ | 102503913​ | 102506146​ | *PAX2​* | 0.00023057​ | 0.000446344​ | 18.84422111​ | Gastrulation​ |
| Chr 14​ | 57134265​ | 57134624​ | *OTX2(dist=131999)​* | 0.000516381​ | 0.000896438​ | -10.79073576​ | Gastrulation​ |
| Chr 9​ | 20097800​ | 20098040​ | *SLC24A2(dist=308765),​*  *MLLT3(dist=243627)​* | 0.000559934​ | 0.000962731​ | -30​ | Sodium/Calcium exchangers; Formation of RNA Pol II elongation complex​ |
| Chr 9​ | 16765846​ | 16766081​ | *BNC2​* | 0.000603434​ | 0.001031208​ | -14.30995475​ | N/A​ |
| Chr 9​ | 80317666​ | 80317885​ | *GNAQ(dist=13139)​* | 0.001129468​ | 0.001779464​ | -6.538461538​ | Signal Transduction​ |
| Chr 11​ | 86641554​ | 86641827​ | *FZD4(dist=14894)​* | 0.001395779​ | 0.002130429​ | -19.66342817​ | Clathrin-mediated endocytosis​ |
| Chr 4​ | 95870482​ | 95870763​ | *BMPR1B​* | 0.00148596​ | 0.002249467​ | -10.30919841​ | Signaling by TGFB family members​ |
| Chr 1​ | 164927838​ | 164928113​ | *LMX1A(dist=242991)​* | 0.001754062​ | 0.002589168​ | -22.00825309​ | N/A​ |
| Chr 20​ | 32856659​ | 32857248​ | *ASIP​* | 0.001770749​ | 0.002610219​ | 11.78146149​ | N/A​ |
| Chr 6​ | 110012365​ | 110012769​ | *FIG4​* | 0.003704376​ | 0.00488562​ | 21.73913043​ | Synthesis of PIPs at the early endosome membrane​ |
| Chr 20​ | 32591467​ | 32591741​ | *RALY​* | 0.003821872​ | 0.005024711​ | 9.409175953​ | N/A​ |
| Chr 6​ | 10415018​ | 10415318​ | *TFAP2A​* | 0.004778043​ | 0.006058563​ | 8.676307008​ | Negative regulation of activity of TFAP2 (AP-2) family transcription factors​ |

**Supplementary Figure 11: Pathway Enrichment Analysis**

A Bar plot showing gene pathway enrichment analyses of genes within *q*–value–significant DMRs at DM >5% (F). Generated using ReactomePA^S7^*.*

**
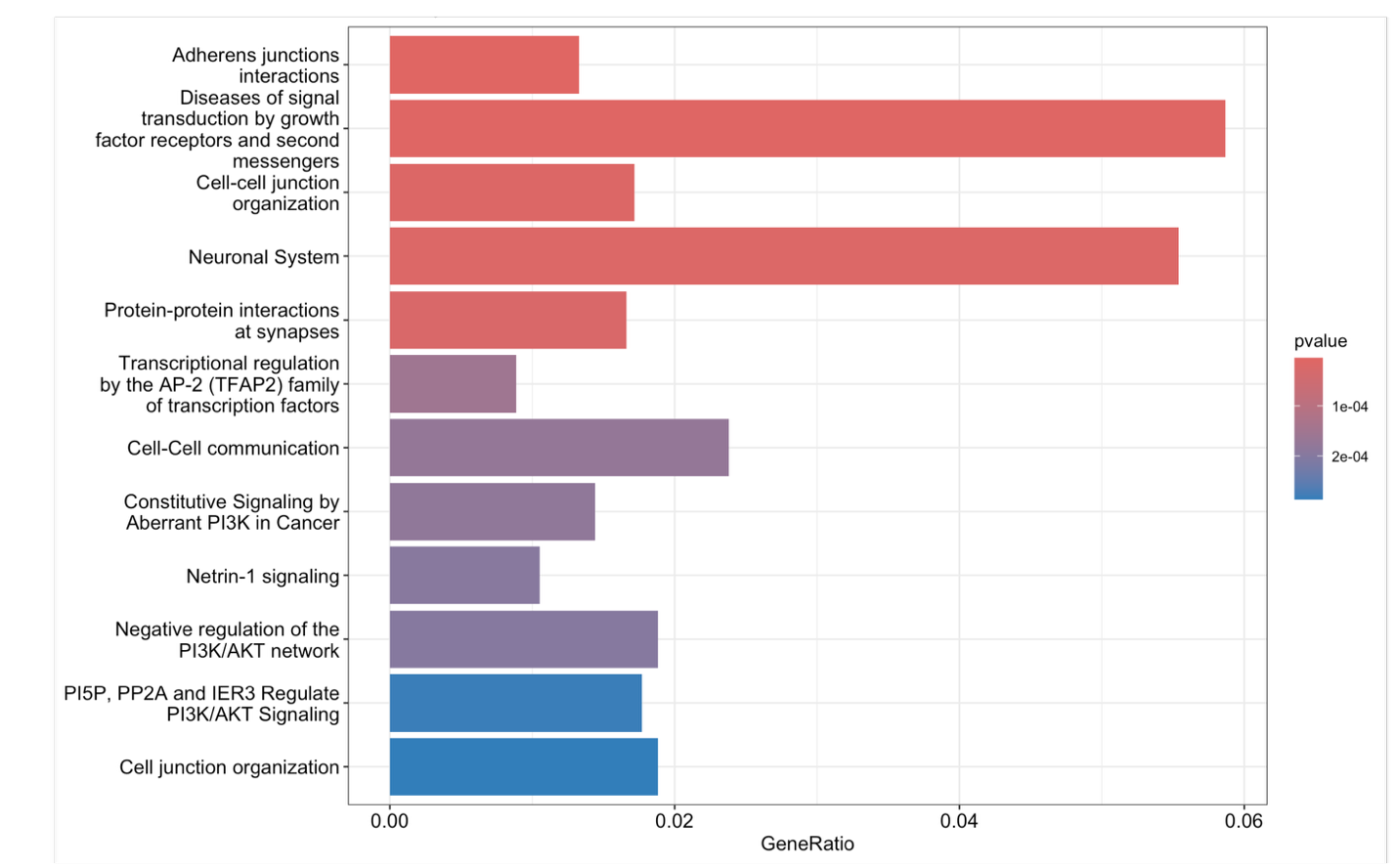
**

**Supplementary References**

S1. Liu, Y., Han, Y., Zhou, L., Pan, X., Sun, X., Liu, Y., Liang, M., Qin, J., Lu, Y., and Liu, P. (2020). A comprehensive evaluation of computational tools to identify differential methylation regions using RRBS data. Genomics *112*, 4567–4576. <https://doi.org/10.1016/j.ygeno.2020.07.032>.

S2. Bergström, A., McCarthy, S.A., Hui, R., Almarri, M.A., Ayub, Q., Danecek, P., Chen, Y., Felkel, S., Hallast, P., Kamm, J., et al. (2020). Insights into human genetic variation and population history from 929 diverse genomes. Science *367*. <https://doi.org/10.1126/science.aay5012>.

S3. Pelegí-Sisó, D., Prado, P. de, Ronkainen, J., Bustamante, M., and González, J.R. (2020). methylclock : a Bioconductor package to estimate DNA methylation age. Bioinformatics *37*, 1759–1760. <https://doi.org/10.1093/bioinformatics/btaa825>.

S4. Moss, J., Magenheim, J., Neiman, D., Zemmour, H., Loyfer, N., Korach, A., Samet, Y., Maoz, M., Druid, H., Arner, P., et al. (2018). Comprehensive human cell-type methylation atlas reveals origins of circulating cell-free DNA in health and disease. Nat. Commun. *9*, 5068. <https://doi.org/10.1038/s41467-018-07466-6>.

S5. IB, G., V, E., and A, A. (2025). deconvR: Simulation and Deconvolution of Omic Profiles. doi:10.18129/B9.bioc.deconvR, https://github.com/BIMSBbioinfo/deconvR/deconvR - R package version 1.12.0, <http://www.bioconductor.org/packages/deconvR>.

S6. Milacic, M., Beavers, D., Conley, P., Gong, C., Gillespie, M., Griss, J., Haw, R., Jassal, B., Matthews, L., May, B., et al. (2023). The Reactome Pathway Knowledgebase 2024. Nucleic Acids Res. *52*, D672–D678. <https://doi.org/10.1093/nar/gkad1025>.

S7. Yu, G., and He, Q.-Y. (2015). ReactomePA: an R/Bioconductor package for reactome pathway analysis and visualization. Mol. Biosyst. *12*, 477–479. https://doi.org/10.1039/c5mb00663e.
